# Striatal neuron dysfunction in C9ORF72-FTD/ALS is driven by AIS and potassium channel dysregulation

**DOI:** 10.1016/j.celrep.2026.117672

**Published:** 2026-07-07

**Authors:** Iris-Stefania Pasniceanu, Manpreet S. Atwal, Cleide Dos Santos Souza, Tobias Moll, Marianne King, Connie Treanor, Daniel Cabezas de la Fuente, Ryan J.H. West, Laura Ferraiuolo, Matthew R. Livesey

**Affiliations:** 1 Sheffield Institute for Translational Neuroscience, Division of Neuroscience, University of Sheffield, Sheffield S10 2HQ, UK; 2 The Neuroscience Institute, University of Sheffield, Sheffield S10 2HQ, UK

**Keywords:** *C9ORF72*, ALS, FTD, striatal, excitability, synaptic, inhibitory, neuron, electrophysiology

## Abstract

Frontotemporal dementia (FTD) and amyotrophic lateral sclerosis (ALS) form a neurodegenerative spectrum characterized by progressive cognitive, behavioral, and motor decline, yet the contribution of the striatum to disease pathophysiology remains poorly understood. Here, we generate inhibitory striatal medium spiny neurons (MSNs) from human induced pluripotent stem cells carrying the *C9ORF72* repeat expansion, the most common genetic cause of FTD/ALS, and compare them with isogenic-corrected, control, and patient-derived motor neurons. Using whole-cell electrophysiology, pharmacological manipulation, and high-resolution imaging, we identify a vulnerability of *C9ORF72* MSNs to develop intrinsic hypoexcitability with linked synaptic dysfunction. These abnormalities are associated with axon initial segment shortening and altered voltage-gated potassium channel function relative to control and isogenic-corrected neurons. Pharmacological modulation partially restores action potential waveform properties, indicating that key electrophysiological abnormalities are reversible. These findings identify the striatum as a critical site of dysfunction in FTD/ALS and highlight striatal excitability as a potential therapeutic target.

## Highlights

• C9ORF72 repeat expansion mutations cause hypoexcitability in striatal neurons
• Axon initial segment shortening impairs spike initiation
• Reduced potassium channel function alters spike repolarization
• Spike timing deficits disrupt inhibitory microcircuit fidelity

## Graphical abstract

**Figure:**
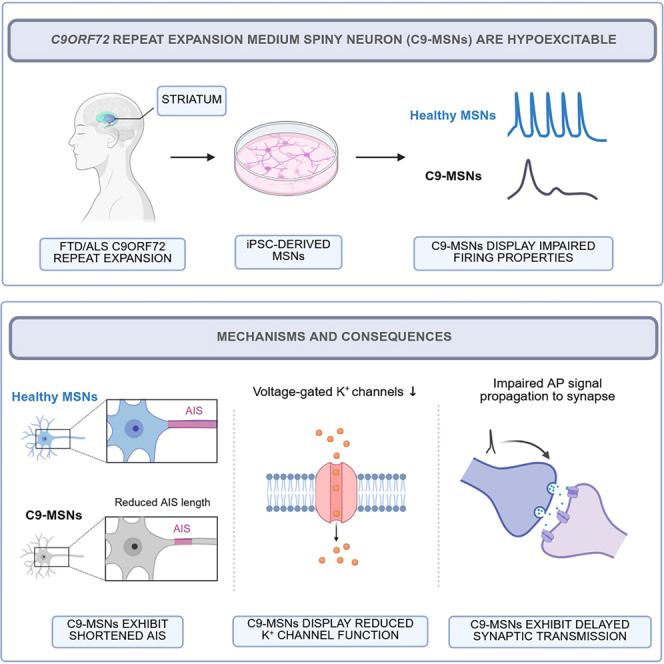

## Teaser

Striatal medium spiny neurons derived from patients with *C9ORF72* repeat expansion mutations show intrinsic hypoexcitability driven by axon initial segment shortening and potassium channel dysregulation. These defects impair spike timing and inhibitory microcircuit precision, identifying striatal dysfunction as a mechanistic contributor to frontotemporal dementia and amyotrophic lateral sclerosis.

## Introduction

Neuronal degeneration in the prefrontal cortex and temporal lobes characterizes frontotemporal dementia (FTD), while motor neuron (MN) loss defines amyotrophic lateral sclerosis (ALS). Despite distinct clinical presentations, FTD and ALS share genetic, pathological, and clinical features, forming a neurodegenerative continuum (FTD/ALS) for which no cure exists. FTD/ALS leads to progressive cognitive and behavioral decline, including apathy, impaired self-awareness, language dysfunction, emotional withdrawal, and executive deficits.^1^ Although traditionally linked to cortical pathology, cognitive deficits in FTD/ALS are now recognized to be more extensive, involving subcortical basal ganglia circuitry.^2^^,^^3^^,^^4^

Functionally, the striatum forms the primary input nucleus of the basal ganglia, receiving excitatory innervation from glutamatergic projection neurons originating in the cortex. These nuclei integrate such information by inhibiting the activity of downstream basal ganglia circuits, including the globus pallidus, substantia nigra, and thalamus, which feedback to cortical areas to regulate behavior. Medium spiny neurons (MSNs) form the principal cell type of the striatum (>90%), are GABAergic in nature, and therefore mediate the main inhibitory output of the striatum.^5^ The striatum is comprised of two anatomically distinct areas: the dorsal striatum, which is primarily involved in motor control, and the ventral striatum, which plays a central role in executive, social, and language functions that align closely with cognitive behaviors that are profoundly impaired in FTD/ALS.^1^^,^^6^ Notably, the frontotemporal cortical neurons projecting to the striatum are a vulnerable population in FTD/ALS, with numerous neuroimaging studies highlighting reduced front-striatal connectivity as a hallmark of disease.^2^^,^^3^^,^^4^ Multiple lines of evidence now suggest the striatum is itself a substrate of FTD/ALS and contributes to disease pathogenesis.

Structural imaging and postmortem studies consistently show marked striatal atrophy and neuronal loss in patients with FTD/ALS, with striatal volume inversely correlating with behavioral symptom severity.^7^^,^^8^^,^^9^^,^^10^^,^^11^^,^^12^^,^^13^ Striatal abnormalities are also evident in presymptomatic individuals, especially *C9ORF72* repeat expansion (*C9ORF72*^*RE*^) carriers, the most common genetic form of FTD/ALS.^8^^,^^9^^,^^11^^,^^14^ Neuropathological studies show TDP-43 aggregates, a hallmark of behavioral variant FTD/ALS, are prominent in both the cortex and subcortical structures, including striatal MSNs and their projections.^15^^,^^16^ Experimental models confirm that TDP-43 pathology in the striatum induces neurodegeneration and cognitive decline.^17^ The ventral striatum, particularly the nucleus accumbens, is notably vulnerable, with pronounced TDP-43 pathology and neuronal loss observed in this region.^2^^,^^8^^,^^10^^,^^13^^,^^15^^,^^18^^,^^19^^,^^20^ Further, as a hub of the mesolimbic pathway, the nucleus accumbens integrates dopaminergic input from the ventral tegmental area and mediates reward processing and motivation; functions disrupted in FTD and previously targeted therapeutically.^21^ Together, these findings underscore the striatum’s involvement in the cognitive and behavioral deficits of FTD/ALS, though striatal dysfunction remains incompletely understood.

Neuronal dysfunction, via altered intrinsic excitability, synaptic transmission, and network activity, plays an early and active role in FTD/ALS pathogenesis by driving neuronal injury, pathological protein accumulation and underpinning cognitive and motor symptoms.^22^ Prior research has primarily focused on cortical and motor circuits. Here we show that GABAergic striatal MSNs derived from *C9ORF72*^*RE*^ patient iPSCs are electro physiologically hypoexcitable. Compared to both healthy and isogenic controls, *C9ORF72*^*RE*^ MSNs exhibit reduced action potential (AP) firing and disturbed post-threshold properties, a phenotype not observed in MNs derived from the same patient lines. *C9ORF72*^*RE*^ MSN disturbances are associated with the dysregulation of potassium channels and axon initial segment dynamics, with partial rescue of AP waveform achievable via potassium channel modulation. We show that this impacts downstream synaptic function in MSNs. Our findings implicate striatal neuron loss-of-function as a feature of *C9ORF72*^*RE*^ FTD/ALS, expanding the scope of functional neuronal disturbances and, thus, therapeutic opportunity beyond cortical and motor regions.

## Results

### C9ORF72^RE^-medium spiny neuron (C9-MSN) generation

**Figure 1:**
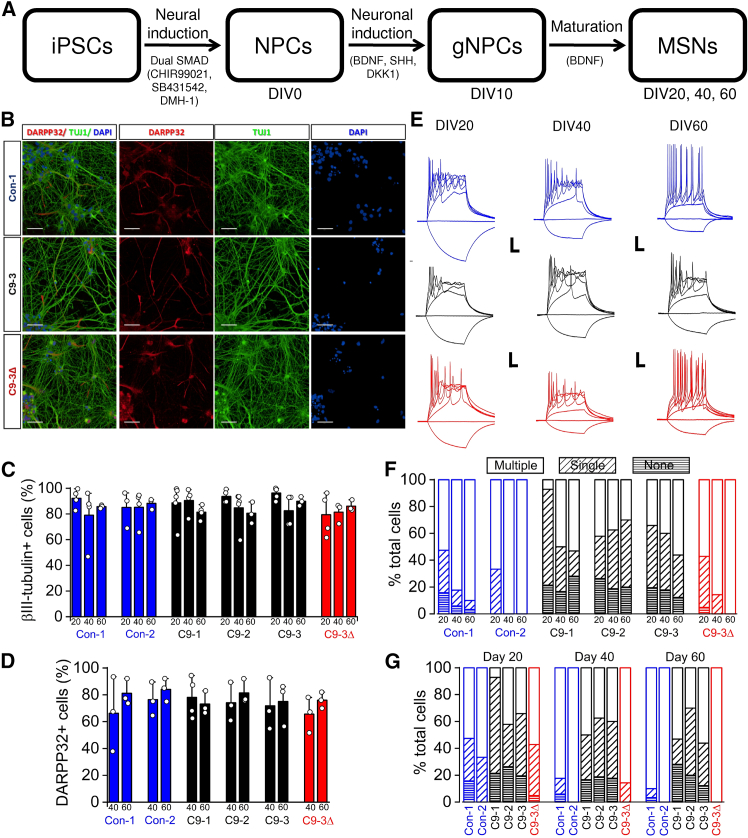
iPSC-derived MSNs (A) Summary diagram of protocol for *in vitro* MSN generation. (B) Representative images of DIV60 Con-1, C9-3 and C9-3Δ MSNs stained for β-III-tubulin (TUJ1) and DARPP-32. Nuclei were counterstained with DAPI. Scale bars, 50 μm. (C) Mean ± SEM percentage of DAPI labeled cells that were β-III-tubulin+ at DIV20-60. (D) Mean ± SEM percentage of β-III-tubulin+ labeled cells that were DARPP-32+ at DIV40 and 60. Data were derived from at least 3 *de novo* preparations. Statistics, one-way ANOVA followed by Tukey’s multiple comparison test. (E) Representative whole-cell current-clamp voltage responses from individual Con-1 (blue), C9-3 (black), and C9-3Δ (red) MSNs at DIV20-60 are shown in response to a train of incremental current injections (−20pA–50pA in 10pA steps) from a holding potential of −84mV. C9-3 MSNs show impaired excitability properties versus Con-1 and C9-3Δ. Note that not all voltage responses from the current protocol are shown for clarity. Scale bars, 20mV, 100ms. (F) Responses of individual MSNs to current stimulation were classified into no response, single Aps, or multiple APs for all MSN lines at DIV20-60. The percentage responsivity of all C9-MSNs shows an impaired firing capability over time, but C9-3Δ, Con-1, and Con-2 MSNs yield robust responses by DIV40. (G) Data in F plotted by DIV, highlighting the reduced comparative responsivity in C9 MSNs at DIV40 and 60. Con-1: *n* = 30–38, *N* = 9–14; Con-2: *n* = 15–21, *N* = 4–10; C9-1: *n* = 6–32, *N* = 3–6; C9-2: *n* = 16–30, *N* = 4–7; C9-3: *n* = 39–41, *N* = 11–12; C9-3Δ: *n* = 20–26, *N* = 8–9.

MSNs were derived *in vitro* from iPSCs using a previously established protocol, which yields highly enriched MSN cultures (23; Figure 1A). The iPSC lines were obtained from two unrelated healthy individuals (Con-1, Con-2), three unrelated individuals carrying the *C9ORF72*^*RE*^ mutation (C9-1, C9-2, C9-3), and one gene-edited line (C9-Δ3), created from C9-3 using CRISPR-Cas9 technology to remove the GGGGCC repeat expansion mutation. The iPSC lines were characterized and described in previous studies (24–27; also see STAR★Methods). In summary, neurogenesis of iPSCs was performed using a dual SMAD inhibition protocol to generate PAX6+ and Nesting+ neuronal progenitor cells (NPCs; Figure S1A). NPCs were then patterned and differentiated toward GABAergic MSNs using BDNF, SHH, and DKK-1. Subsequent maturation of MSNs was achieved through BDNF supplementation. Immunocytochemical characterization was conducted at 20, 40, and 60 days *in vitro* (DIV) post NPC generation to assess the efficiency of MSN differentiation. By DIV20, 75–90% of cells across all lines displayed neuronal morphology and were β-III-tubulin positive, a post-mitotic neuronal marker that remained consistent through to DIV60 (Figures 1B and 1C). The high efficiency of neuronal differentiation across all iPSC lines employed was further confirmed by the negligible presence (<5%) of the astrocytic marker GFAP throughout the culture period (Figure S1B) and the low expression (<5%) of the progenitor marker Nesting by DIV20 (Figure S1C). Expression of DARPP-32, a mature striatal neuron marker, became prominent at DIV40 in all lines and was present in approximately 70–80% of β-III-tubulin+ neurons by DIV60 (Figures 1B and 1D), comparable to previously reported efficiencies.^23^ MSNs are GABAergic neurons, thus we confirmed positive detection of neurotransmitter GABA in 75–85% of DIV60 β-III-tubulin+ neurons across all lines (Figures S1D and S1E). To assess the potential for enhanced cell death of C9-MSNs versus control and C9-3Δ MSNs, we measured active caspase-3 expression, an apoptotic marker, and found no significant differences across DIV60 cultures from all lines (Figure S1F). All iPSC lines efficiently differentiated into enriched cultures of viable DARPP-32+, GABA+ MSNs.

### C9-MSNs display intrinsic hypoexcitability

Since striatal performance in FTD/ALS is consistent with a loss-of-function,^2^^,^^3^ we examined whether C9-MSNs exhibited neurophysiological impairments by performing whole-cell patch-clamp electrophysiology, allowing for detailed evaluation of MSN function at the single cell level. To assess AP generation, MSNs were depolarized using a sequential current stimulation protocol ranging from −20 to +50 pA from a holding potential of −84 mV (including liquid junction potential of −14 mV). MSNs for each line were assessed at DIV20, 40, and 60 to assess the time course of excitability. Representative voltage responses evoked by the protocol (Figure 1E) and categorization of the responses of individual MSN responses into three distinct behaviors: multiple APs (≥2), single AP, or no response, demonstrate a progressive maturation toward higher levels of excitability with time for each line (Figure 1F). By DIV40, however, C9-MSNs showed a notably reduced responsiveness compared to healthy and isogenic controls, with ∼50–65% of neurons firing only a single AP or remaining unresponsive, a deficit that persisted through to DIV60 (Figure 1G), indicating impaired intrinsic responsiveness.

**Figure 2:**
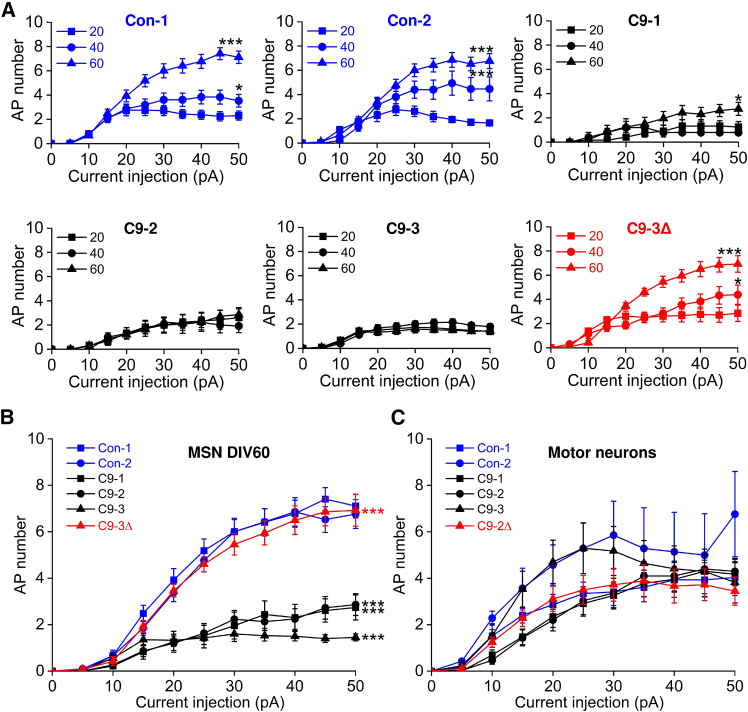
C9-MSNs are hypoexcitable (A) Mean ± SEM current input-AP number output relationships for each MSN line showing the development of excitability from DIV20-60. C9-MSN excitability does not develop over time. (B) Mean ± SEM comparative DIV60 data for all lines show C9-MSNs have a significantly reduced capacity to fire APs versus healthy controls, and, that C9-3Δ MSNs have a significantly higher capacity to fire APs versus C9-3 MSNs. Data: Con-1: *n* = 27–36, *N* = 9–14; Con-2: *n* = 15–21, *N* = 4–10; C9-1: *n* = 5–23, *N* = 2–6; C9-2: *n* = 13–24, *N* = 4–7; C9-3: *n* = 33–36, *N* = 11–12; C9-3Δ: *n* = 20–26, *N* = 8–9. (C) Mean ± SEM current input-AP number output relationships from DIV40 MNs generated from the same C9 and control iPSC lines in B show no excitability differences. Supporting data for MNs are presented in Figure S3. Note that the C9-2Δ line was employed because the C9-3Δ line did not efficiently differentiate into MNs. Data: Con-1: *n* = 29, *N* = 7; Con-2: *n* = 7, *N* = 2; C9-1: *n* = 23, *N* = 7; C9-2: *n* = 30, *N* = 9; C9-2Δ: *n* = 17, *N* = 4; C9-3: *n* = 17, *N* = 3. Statistics, ^∗^*p* < 0.05 and ^∗∗∗^*p* < 0.001 from two-way repeated measures ANOVA followed by post hoc comparisons using estimated marginal means with Tukey’s correction.

To examine excitability, current stimulus input – AP output relationships were constructed for MSNs for all lines (Figure 2A). As expected from the neurophysiological maturation of iPSC-derived MSNs,^24^ the number of APs produced by current depolarization increased progressively and significantly over time in Con-1 and Con-2 MSNs. In contrast, all C9-MSN lines consistently exhibited low AP output at all time points, which failed to significantly progress with time in two of the lines and significantly, but only modestly, in C9-1 MSNs. The C9-3Δ MSNs mirrored the healthy controls, demonstrating a significantly increased AP output over the culture period. Critically, all C9-MSN lines at DIV60 comparatively showed significantly reduced input-output relationships for equivalent levels of current stimulation versus control MSNs, indicating a reduced capacity for AP generation relative to controls. Further, MSNs derived from the isogenic C9-3Δ line displayed a significantly improved AP output profile compared to C9-3, which is not significantly different from the healthy control MSNs (Figure 2B). These findings show that C9-MSNs develop a persistent hypoexcitability phenotype over time.

To determine whether C9-MSN dysfunction is selective to excitability properties, we then examined other key functional properties of MSNs. Patients with FTD/ALS and models of FTD/ALS show glutamatergic and GABAergic transmission deficits, which thereby represent key therapeutic targets for disease.^21^^,^^25^ Glutamatergic neurons, which are a primary vulnerable population in FTD/ALS, project from the prefrontal cortex to drive MSN excitability and regulate FTD/ALS-impacted cognitive behaviors.^26^ Further, MSNs receive GABAergic input that regulates MSN inhibition via GABA_A_ receptor expression. Changes in excitability can also be driven via the loss of transmitter-gated AMPA, GABA_A_, and NMDA receptor ion channels on target neurons,^25^ potentially including MSNs. However, by assessing pharmacological responsiveness of MSNs to selective receptor agonists, we determined that the functional expression of these ionotropic receptors were not impacted in C9-MSNs (Figure S2), collectively indicating the hypoexcitability presented by C9-MSNs is independent of functional AMPA, GABA_A_ and NMDA receptor defects. Given that these receptors functionally increase with MSN development,^27^^,^^28^ the data indicate that hypoexcitability in C9-MSNs is unlikely to stem from a broader mechanism of impaired cellular development.

Before investigating the mechanisms underlying hypoexcitability in C9-MSNs, we assessed whether C9-MSNs have a specific vulnerability to developing a hypoexcitable phenotype compared to other implicated cell types in ALS. To do so, we examined the excitability of lower MNs derived from the same patient iPSC lines. Previously, these lines were shown to efficiently differentiate into β-III-tubulin+ neuronal cultures, primarily consisting of SMI-32+ and CHAT+ MNs,^29^^,^^30^ following an established protocol.^31^ We determined that the excitability of *C9ORF72*^*RE*^ DIV40 MNs was not significantly different from control or isogenic MNs (Figures 2C; Figure S3). This is consistent with previous studies that show the excitability of *C9ORF72*^*RE*^ MNs remains unaffected versus control MNs in highly enriched MN-cultures.^22^^,^^32^^,^^33^ Collectively, these data show that the *C9ORF72*^*RE*^ genetic background specifically induces vulnerability to the development of intrinsic hypoexcitability in MSNs.

### The action potential waveform is disturbed in C9-MSNs

Intrinsic hypoexcitability typically results from defects in cellular mechanisms regulating AP generation at the post- and/or sub-threshold level. First, we considered possible sub-threshold impairments, including the input resistance (R_IN_), a major parameter controlling the responsiveness of neurons to stimulation, along with the resting membrane potential (RMP) and whole-cell capacitance, key parameters indicative of mechanisms required for maintaining excitability and cell membrane integrity, respectively. Furthermore, such parameters are reflective of MSN development, i.e., decreasing R_IN_, hyperpolarizing RMP, and increasing whole-cell capacitance.^34^ These parameters showed equivalent development with time across all lines with no significant comparative differences between lines (Figure S4). Altered sub-threshold properties are therefore not contributors to C9-MSN hypoexcitability and, additionally, provide supporting evidence of the causal mechanism not being due to developmental impairments.

**Figure 3:**
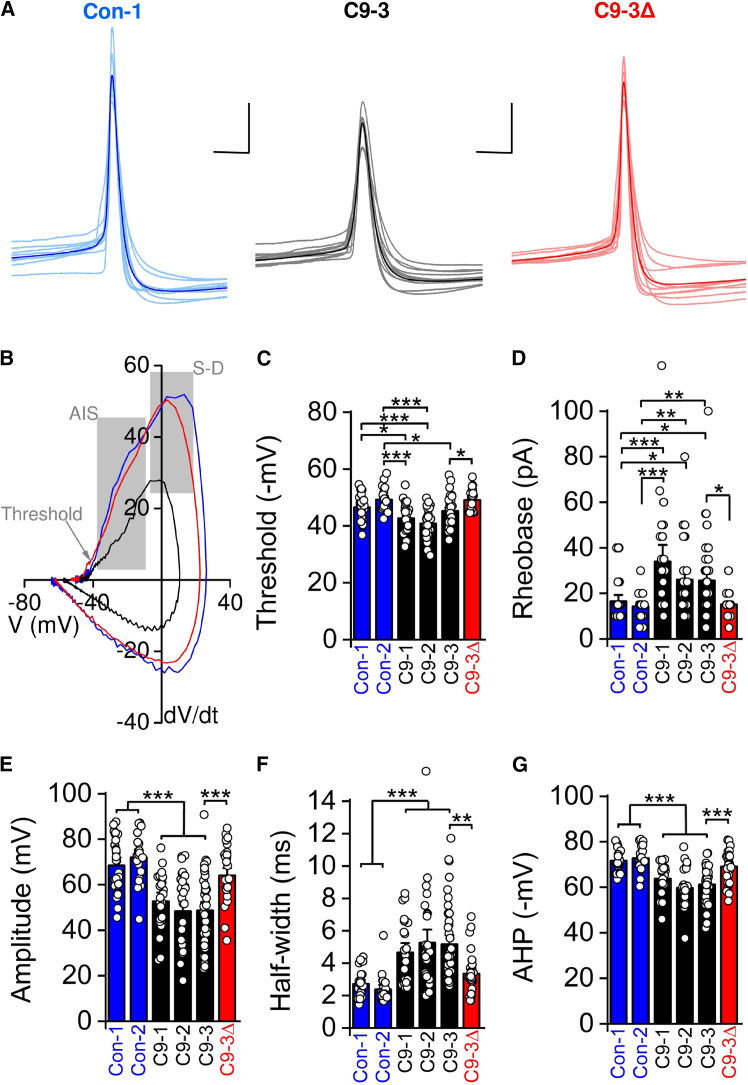
C9-MSNs show impaired AP waveform (A) Example and mean DIV60 AP waveforms for Con-1 (blue), C9-3 (black), and C9-3Δ (red) evoked by the rheobasic current. The mean AP waveforms for each group of recordings are shown in bold color, while individual AP traces are in a lighter color shade (10 single AP waveforms are shown for clarity). The traces are aligned to 0 mV-crossing time. Note the increased duration and reduced amplitude of the AP waveform for C9-3 MSNs versus Con-1 and C9-3Δ. Scale bars, for APs, 20 mV, 10 ms. (B) Phase plot analysis of AP waveforms presented in A shows a disturbed C9-MSN waveform, displaying reduced AIS and somatodendritic (S-D) peak components in the depolarization phase and impaired repolarization. (C–G) Mean ± SEM specific AP parameters were measured for all MSNs, including threshold, the minimum current to evoke an AP (rheobase), amplitude, duration (half-width), and afterhyperpolarization (AHP). Statistics, ^∗^*p* < 0.05, ^∗∗^*p* < 0.01, and ^∗∗∗^*p* < 0.001 from one-way ANOVA followed by Tukey’s multiple comparisons test. Data numbers as presented in Figure 2. Supporting data in Figure S5.

We next examined potential disturbances related to AP generation. Averaged waveforms (Figure 3A) demonstrate that C9-MSNs exhibited a visibly slower and blunted AP waveform compared to healthy and isogenic controls, consistent with a reduced capacity to fire APs. Waveforms were further analyzed using phase-plane analysis, which provides a dynamic assessment of AP kinetics, including spike initiation driven by voltage-gated sodium channel (NaV) activation, AP amplitude, and repolarization mediated by voltage-gated potassium (Kv) channels (Figure 3B), thereby offering mechanistic insight into neuronal excitability and highlighting the differences in the waveforms across the lines.

We have determined that AP initiation is altered in C9-MSNs. C9-MSNs exhibited a significantly depolarized average AP threshold compared to control and isogenic MSNs, indicating a higher depolarization requirement for AP initiation (Figure 3C). Directly consistent with this, the minimum amount of depolarizing current required to initiate AP firing, the rheobase, was significantly increased in C9-MSNs versus control and isogenic MSNs (Figure 3D). Further phase-plane analysis revealed that the initial slope of the AP depolarizing phase was reduced in C9-MSNs, indicating impaired efficiency in NaV activation. Given that recordings were performed at the soma, the depolarizing phase reflects the activation of both NaVs in the AIS and somatodendritic space (Figure 3B). C9-MSNs exhibited reduced dV/dt peaks in both components, indicating impaired spike initiation at the AIS and reduced propagation to the soma. In line with these deficits, AP amplitude in C9-MSNs was significantly reduced versus control and isogenic MSNs (Figure 3E). Together, C9-MSNs show dysregulation in AP initiation, potentially due to NaV and/or AIS dysregulation.

We next analyzed the repolarization phase of the AP, which reflects Kv channel activity. C9-MSNs displayed a markedly reduced negative dV/dt peak compared to control and isogenic lines, indicating impaired repolarization kinetics (Figure 3B). This was further supported by half-width data, a measure of the spike duration, where C9-MSNs displayed significantly prolonged half-widths (∼double) versus control and isogenic MSNs (Figure 3F). Moreover, the afterhyperpolarization (AHP), also determined by Kv channel function, was significantly more depolarized in C9-MSNs versus control and isogenic MSNs (Figure 3G). As AP repolarization is dependent on the timely activation of voltage-gated Kv channels, the observed deficits may result from impaired AP initiation leading to suboptimal Kv activation. However, this may also be due to other mechanisms leading to a loss-of-function of Kv channels. Thus, these findings point to disrupted Kv channel regulation as a contributing factor to altered AP waveform properties in C9-MSNs.

Collectively, these findings show that hypoexcitability in C9-MSNs is due to the disruption of post-threshold AP dynamics in C9-MSNs, consistent with Kv and AIS/NaV channel dysregulation. Moreover, the deficits in C9-MSN AP waveform described above develop over the course of culture duration, consistent with reduced C9-MSN firing (Figure S5). Further, we show that AP waveform properties were not detected in *C9ORF72*^*RE*^ MNs (Figure S3), reinforcing the vulnerability of the development of hypoexcitability in C9-MSNs.

### Ion channel function is impaired in C9-MSNs

**Figure 4:**
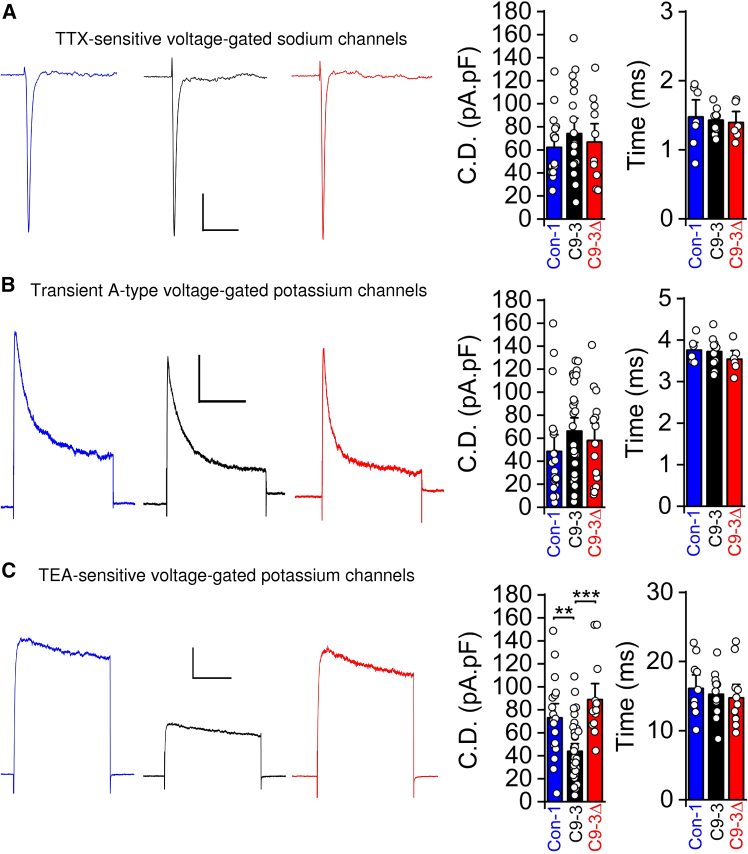
Slow-activated potassium channel dysfunction in C9-MSNs (A–C) Example pharmacologically isolated whole-cell voltage-clamp NaV, I_A_, and slow-activated Kv channel currents, respectively, from DIV60 Con-1 (blue), C9-3 (black), C9-3Δ (red) MSNs. Scale bars, NaV (250 pA, 10 ms); I_A_ (200pA, 200 ms); slow-activated Kv (500pA, 150 ms). See STAR★Methods for full leak subtraction protocol details for each ion channel family. The mean ± SEM current density (C.D., whole-cell current amplitude/whole-cell capacitance) shows that slow-activated Kv channel, but not NaV or I_A_, function is significantly reduced in C9-3 versus Con-1 MSNs and C9-3Δ. NaV data: Con-1, *n* = 16, *N* = 4; C9-3, *n* = 19, *N* = 4; C9-3Δ, *n* = 11, *N* = 4. I_A_ data: Con-1, *n* = 20, *N* = 5; C9-3, *n* = 27, *N* = 5; C9-3Δ, *n* = 15, *N* = 5. Slow-activated Kv data: Con-1, *n* = 18, *N* = 4; C9-3, *n* = 34, *N* = 5; C9-3Δ, *n* = 13, *N* = 3. The mean ± SEM rise time from 5% baseline to 95% peak amplitude of current, as a measure of the activation kinetics, is presented for each of the NaV, I_A_, and slow-activated Kv channel currents and does not yield any differences between MSN lines. NaV data: Con-1, *n* = 7, *N* = 2; C9-3, *n* = 10, *N* = 2; C9-3Δ, *n* = 6, *N* = 2. I_A_ data: Con-1, *n* = 6, *N* = 5; C9-3, *n* = 13, *N* = 5; C9-3Δ, *n* = 6, *N* = 5. Slow-activated Kv data: Con-1, *n* = 10, *N* = 3; C9-3, *n* = 11, *N* = 5; C9-3Δ, *n* = 11, *N* = 3. Statistics, ^∗∗^*p* < 0.01 and ^∗∗∗^*p* < 0.001 from one-way ANOVA followed by Tukey’s multiple comparisons test.

To investigate whether the altered AP dynamics in C9-MSNs reflect changes in NaV and Kv channel function, we performed whole-cell voltage-clamp recordings designed to isolate and characterize these conductance’s. NaV-mediated currents were pharmacologically isolated using tetrodotoxin (TTX) in conjunction with a leak subtraction protocol (Figure 4A; protocol described in detail in Methods). From these isolated currents, we determined the current density, a measurement of functional NaV channel expression normalized for differences in cell membrane size (whole-cell capacitance), and the time-to-peak, an assessment of the biophysical activation and inactivation kinetics of NaV channels. Despite significant alterations in AP threshold, depolarization and amplitude observed in current-clamp recordings (Figure 3), both NaV current density and kinetic properties were unchanged in C9-MSNs compared to healthy and isogenic controls (Figure 4A). This apparent paradoxical discrepancy reflects methodological distinctions between current- and voltage-clamp techniques. In voltage-clamp, recordings from the soma primarily capture NaV currents from the somatodendritic compartment, whereas AP initiation in current-clamp is predominantly driven by NaV channels at the AIS. In this regard, while somatodendritic NaV current density appears preserved in voltage-clamp, phase-plane analysis in current-clamp reveals substantial reductions in somatodendritic dV/dt peaks in C9-MSNs. This contrast indicates that backpropagation of the AP from the AIS to the soma is functionally impaired, indicating an electrical decoupling between these compartments. These data support the interpretation that AP initiation deficits in C9-MSNs arise from AIS-specific dysfunction rather than alterations in global NaV channel biophysics.

Given the observed alterations in the AP repolarization phase of C9-MSNs, specifically AP duration and AHP, we next investigated the potential involvement of Kv channel dysfunction, in addition to adverse activation via impaired AP initiation. Two major Kv families regulate the AP repolarization phase; fast-inactivating A-type (I_A_), and, slow-activating delayed-rectifier and calcium-activated Kv channels, which are activated by the depolarizing AP upstroke. First, using a biophysical leak subtraction approach (see STAR★Methods), we assessed I_A_ currents and found no significant differences in current density or kinetic properties in C9-MSNs (Figure 4B). However, pharmacologically (tetraethylammonium, TEA) isolated slow-activated Kv channel currents (Figure 4C; STAR★Methods) revealed a significant decrease in current density in C9-MSNs compared to controls, but no change in channel kinetics, indicating a loss of functional slow-activated Kv channel function (Figure 4C). These findings are consistent with reduced functional expression of slow-activated Kv channels contributing to the impaired repolarization observed in C9-MSNs.

**Figure 5:**
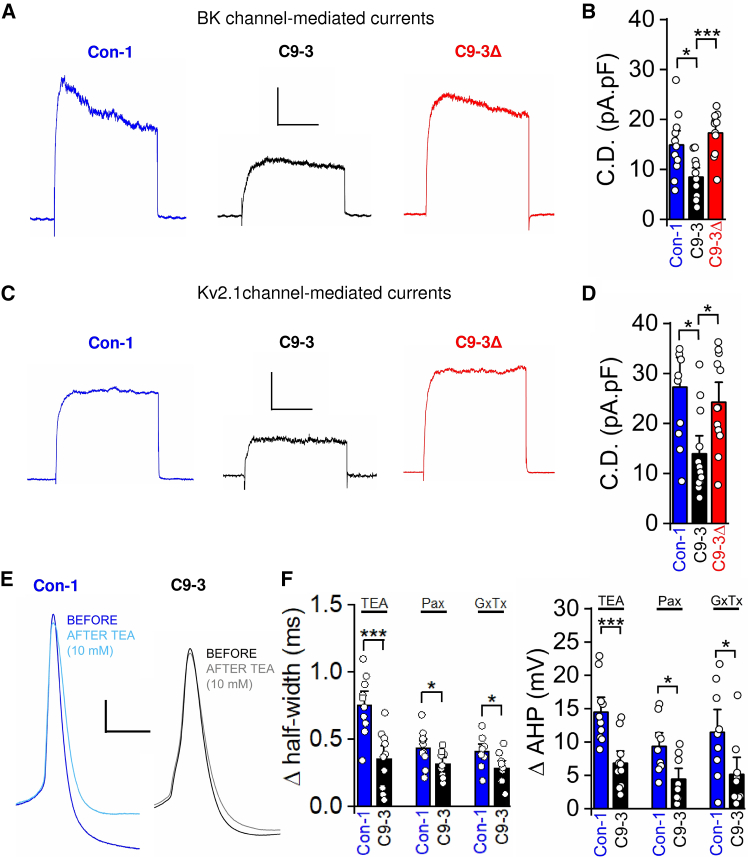
BK and Kv2.1 channel dysfunction in C9-MSNs (A) Example pharmacologically (paxillin) isolated whole-cell voltage-clamp BK channel currents, respectively, from Con-1 (blue), C9-3 (black), and C9-3Δ (red) MSNs. The leak subtraction protocol employed was the same as that used for slow-activated Kv channels using TEA, though this time using paxillin (10 μM). Scale bars, 100pA, 100 ms. (B) The mean ± SEM current density (C.D.) shows that functional BK channel expression is reduced in C9-3 versus Con-1 and C9-3Δ MSNs. Data: Con-1, *n* = 10, *N* = 2; C9-3, *n* = 10, *N* = 2; C9-3Δ, *n* = 10, *N* = 2. Statistics, ^∗^*p* < 0.05 and ^∗∗∗^*p* < 0.001 from one-way ANOVA followed by Tukey’s multiple comparisons test. (C) Example Guangxitoxin-1E (100 nM) isolated whole-cell voltage-clamp Kv2.1 channel currents, respectively, from Con-1 (blue), C9-3 (black), C9-3Δ (red) MSNs using the same leak subtraction protocol employed above. Scale bars, 100pA, 100 ms. (D) The mean ± SEM current density (C.D.) shows that Kv2.1 channels are reduced in C9-3 versus Con-1 and C9-3Δ MSNs. Data: Con-1, *n* = 10, *N* = 2; C9-3, *n* = 12, *N* = 2; C9-3Δ, *n* = 12, *N* = 2. Statistics, ^∗∗^*p* < 0.05, from one-way ANOVA followed by Tukey’s multiple comparisons test. (E) Example AP waveforms before and after the addition of TEA (10 mM, lighter color shade) for DIV60 Con-1 (blue) and C9-3 (black) MSNs. Note the reduced impact of TEA on C9-3 MSNs, consistent with less slow-activated Kv channel function. Scale bars, 20 mV, 10 ms. (F) Mean ± SEM change (Δ) in AP duration (half-width), and AHP, respectively, induced by TEA (Data: Con-1, *n* = 10, *N* = 2; C9-3, *n* = 10, *N* = 2), paxillin (Data: Con-1, *n* = 12, *N* = 2; C9-3, *n* = 10, *N* = 2) and Guangxitoxin-1E (Data: Con-1, *n* = 9, *N* = 2; C9-3, *n* = 9, *N* = 2). Note the reduced shift in these parameters in C9-3 MSNs versus Con-1 MSNs is consistent with reduced slow-activated Kv channel function. Statistics, ^∗^*p* < 0.05 and ^∗∗∗^*p* < 0.001 from *t* test.

To identify specific post-threshold-activated slow-activated Kv channels that may be functionally impaired in C9-MSNs, we initially focused on TEA-sensitive large-conductance calcium-activated potassium (BK) channels that are prominently expressed in the somatodendritic compartments of MSNs and are well-established regulators of AP repolarization dynamics.^35^^,^^36^ To assess BK channel function, we used the selective BK channel blocker paxillin. Pharmacological isolation using leak-subtracted protocols revealed significantly reduced BK-channel current density in C9-MSNs compared to controls (Figures 5A and 5B), indicating reduced functional expression of BK channels in C9-MSNs. We additionally looked at BK channel dysfunction in C9-MNs but did not see a difference in the functional expression versus controls (Figure S3J), confirming that C9-MSNs have a greater vulnerability to developing hypoexcitability.

Notably, reduced BK channel activity has previously been associated with enhanced neuronal excitability,^35^ which contrasts with the hypoexcitability observed in C9-MSNs. This prompted us to examine whether additional slow-activated Kv conductance’s were altered. Kv2.1 channels are TEA-sensitive delayed-rectifier Kv channels highly expressed in MSNs and are known regulators of AP repolarisation.^35^^,^^37^ Similar to BK channels, reduced Kv2.1 function has been associated with increased excitability.^35^ Using a leak-subtraction protocol analogous to that employed for BK currents, we isolated Kv2.1-mediated currents using the selective blocker Guangxitoxin-1E. Kv2.1 current density was significantly reduced in C9-MSNs compared to controls (Figures 5C and 5D). Together, these findings indicate coordinated reduction of multiple slow-activating Kv conductance’s in C9-MSNs, consistent with a broader remodeling of repolarizing mechanisms that contributes to altered spike waveform repolarization dynamics.

To directly test whether reduced slow-activated Kv conductance’s contribute to the altered repolarization phenotype in C9-MSNs, we examined the effects of broad and selective slow-activated Kv channel blockade on AP waveform properties in both control and C9-MSNs. We reasoned that if diminished slow-activated Kv conductance’s underlie the prolonged AP duration and altered AHP observed in C9-MSNs, then pharmacological blockade of slow-activated Kv channels in control MSNs should shift AP waveform parameters toward a C9-like phenotype. Conversely, if slow-activated Kv conductance’s are already reduced in C9-MSNs, additional channel blockade would be expected to produce attenuated effects. Application of TEA (broad slow-activated Kv blocker), paxillin (BK channel blocker), or Guangxitoxin-1E (Kv2.1 blocker) to control MSNs significantly prolonged half-width and depolarized the AHP. Notably, TEA application produced waveform changes in controls that closely resembled those observed in untreated C9-MSNs (Figures 5E and 5F). In contrast, application of the same blockers to C9-MSNs produced significantly smaller changes in half-width and AHP compared to controls. This occlusion effect is consistent with reduced functional slow-activated Kv conductance in C9-MSNs. Together, these findings functionally validate a broad reduction in slow-activated Kv channel activity as a key contributor to impaired repolarization dynamics in C9-MSNs.

Collectively, these findings suggest that the AP firing and waveform abnormalities observed in C9-MSNs are best explained by a combination of AP initiation problems, related to AIS dysfunction, and broadly reduced slow-activated Kv channel function, impairing AP repolarization.

### AIS impairments and rescue of AP waveform in C9-MSNs

**Figure 6:**
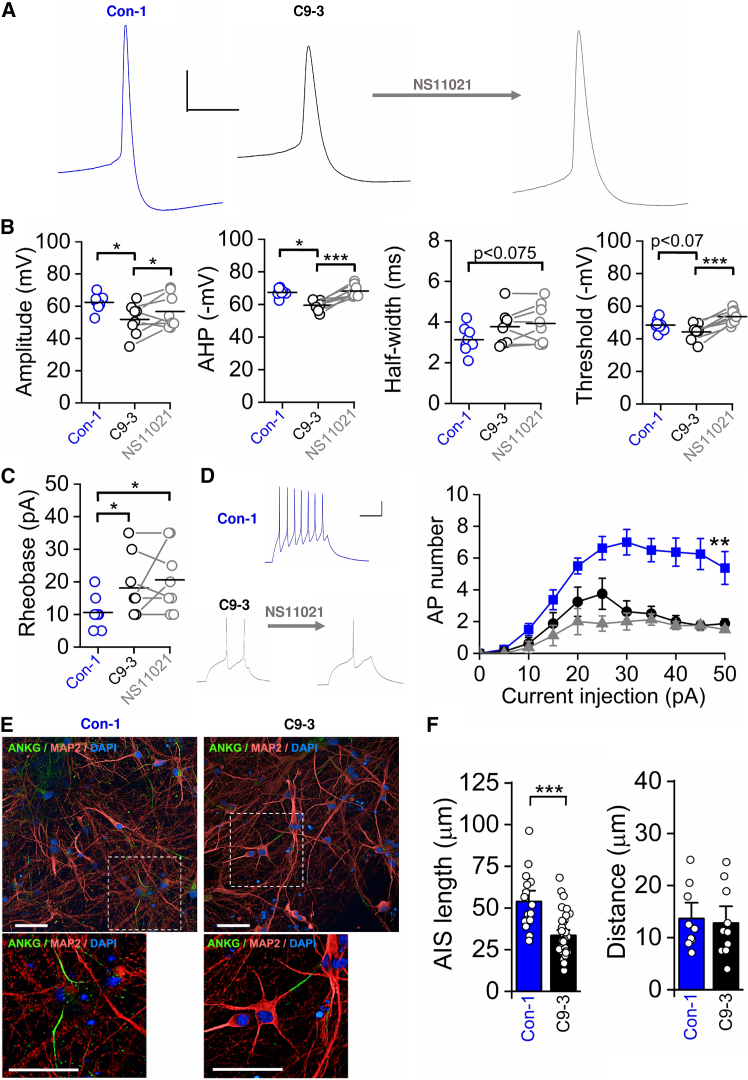
Waveform rescue and impairment of AIS in C9-MSNs (A) Example AP waveforms for Con-1 (blue) and C9-3 MSNs (black) before and after the addition of BK channel activator NS11021 (10 μM, lighter color shade). Scale bars, 20 mV, 10 ms. (B and C) Mean ± SEM AP amplitude, AHP, half-width, threshold, and rheobase of C9-3 MSNs in the presence and absence of NS11021, along with Con-1 MSNs, were examined in parallel. Statistics, ^∗^*p* < 0.05 and ^∗∗∗^*p* < 0.001, from paired *t test* or *t test*. Data: con-1: *n* = 8, *N* = 3. C9-3: *n* = 10, *N* = 4. (D) NS11021 treatment had no effect on AP output in C9-3 MSNs evoked from current stimulation. Con-1 patched in parallel, shown for reference. Data: Con-1: *n* = 8, *N* = 3. C9-3: *n* = 10, *N* = 4. Statistics, two-way repeated measures ANOVA followed by Tukey’s multiple comparisons test. (E) Images of Con-1 and C9-3 stained with AIS marker ankyrin-G, neuronal marker MAP2, and DAPI. Scale bars, 50 μm. (F) Mean ± SEM AIS length and distance of start of AIS from the cell soma. Data: Con-1: *n* = 16, *N* = 4. C9-3: *n* = 36, *N* = 4. ^∗∗∗^*p* < 0.001, *t* test.

Given the pronounced AP waveform abnormalities in C9-MSNs and the functional contribution of BK channel dysfunction, we next evaluated whether enhancing BK channel activity could ameliorate these defects. While no selective Kv2.1 channel potentiators are currently available, BK channel function can be enhanced using NS11021, a selective positive modulator that facilitates BK channel activation at more hyperpolarized membrane potentials. Application of NS11021 (10 μM) to C9-MSNs significantly improved AP waveform characteristics, including increased AP amplitude and hyperpolarization of the AHP phase (Figures 6A and 6B). Surprisingly, AP duration (half-width) remained unchanged following NS11021 treatment (Figure 6B). This apparent lack of effect on AP duration despite AP waveforms clearly being sharpened (Figure 6A) likely reflects the concurrent increase in AP amplitude, which shifts the voltage at which half-width is measured to a more hyperpolarized level, thereby yielding a similar half-width value despite changes in waveform dynamics. NS11021 also hyperpolarized the AP threshold in C9-MSNs, suggesting that enhanced AHP may promote NaV channel recovery from inactivation, increasing their availability, a mechanism likely to be driving the observed increase in AP amplitude (Figure 6B). However, rheobase remained elevated, indicating that subthreshold excitability was not significantly altered despite improvements in AP waveform properties (Figure 6C). Together, these findings demonstrate that the selective activation of BK channels can partially rescue AP waveform deficits in C9-MSNs, offering mechanistic insight and potential therapeutic direction for restoring striatal neuron function in FTD/ALS.

Given the beneficial effects of NS11021 on AP waveform properties, we next investigated whether enhancing BK channel activity could improve current input-AP output relationships in C9-MSNs. BK channel modulation has been closely linked to firing frequency, and activation is often associated with reduced excitability, though its effects are known to be context dependent.^38^ Despite the improvement in AP waveform, NS11021 treatment did not restore firing frequency in C9-MSNs (Figure 6D). This dissociation between waveform rescue and firing behavior suggests the presence of an upstream rate-limiting factor that constrains excitability, independent of AP repolarization kinetics. Further, given the association of reduced BK channel activity with increased neuronal activity, these data support an adaptive response of BK channel activity in response to the upstream rate-limiting factor.

The AIS is a critical site for AP initiation, functioning as a platform for the high-density clustering of NaV and Kv channels. Ankyrin-G is a core scaffolding protein required for AIS formation and ion channel localization and demarcates the AIS. To investigate whether AIS abnormalities contribute to impaired excitability in C9-MSNs, we performed immunostaining for Ankyrin-G. Quantitative analysis revealed a significant reduction in AIS length, but not distance away from the soma, in C9-MSNs compared to control MSNs (Figures 6E and 6F), indicating a structurally diminished AP initiation site, confirming the phase plot data. Given that shorter AIS length is well established to be associated with reduced excitability,^39^ these findings support the hypothesis that AP initiation is compromised in C9-MSNs due to AIS disruption, which likely accounts for the sustained reduction in firing frequency (hypoexcitability) despite improved AP waveform properties in the presence of NS11021.

### Temporal fidelity of synaptic transmission is disrupted in C9-MSN networks

MSNs form recurrent inhibitory microcircuits that are critical for shaping striatal output that underlies behavioral and cognitive function.^40^^,^^41^ Because AP initiation at the AIS determines both the timing and reliability of neurotransmitter release, we hypothesized that the observed shortening of the AIS and slowing of AP kinetics in C9-MSNs would impair the temporal precision of synaptic transmission.

**Figure 7:**
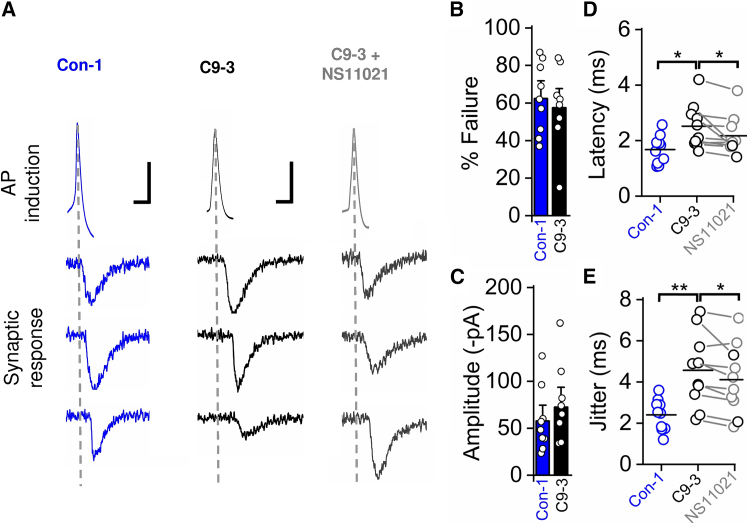
Impaired synaptic function in C9-MSNs (A) Representative recordings from synoptically connected MSN pairs in DIV53-65 Con-1, C9-3, and C9-3 subsequently treated with NS11021. Presynaptic neurons were recorded in current-clamp mode and evoked to fire a single AP with a 50 pA current injection (top traces). Corresponding postsynaptic responses recorded in voltage-clamp (−70 mV) are shown later in discussion. Three representative inhibitory postsynaptic currents from the same pair are displayed to illustrate the fastest (top), median (middle), and slowest (lower) synaptic responses observed across at least 10 trials. C9-3 MSN pairs exhibit prolonged synaptic latency and increased variability (jitter) compared to controls. NS11021 treatment partially normalized these temporal properties. Scale bars, 50 pA/20 mV, 5 ms. (B) Synaptic failure rate, expressed as the percentage of presynaptic APs that did not evoke a detectable postsynaptic response. (C) Peak amplitude of evoked current responses in connected MSN pairs. (D) Synaptic latency, measured as the interval between the peak of the presynaptic AP (*dashed line*) and the onset of the postsynaptic current, defined as the time from the peak of the AP to 5% of the synaptic amplitude. Latency was significantly prolonged in C9-MSNs and partially restored by NS11021. (E) Synaptic jitter, defined for each connected pair as the range between the fastest and slowest evoked responses across at least 10 trials. Jitter was increased in C9-MSNs and partially reduced following NS11021 treatment. Statistics, ^∗^*p* < 0.05 and ^∗∗^*p* < 0.01, from paired *t test* or *t test*. Data: con-1: *n* = 10, *N* = 3. C9-3: *n* = 10, *N* = 3.

To determine whether intrinsic AP abnormalities translate into altered functional connectivity, we performed simultaneous paired whole-cell recordings from neighboring MSNs. The presynaptic neuron was recorded in current-clamp mode and stimulated to fire single APs, while the postsynaptic neuron was voltage-clamped at −70 mV using a CsCl-based internal solution to isolate synaptic currents (Figure 7A). Synaptic currents were sensitive to the GABA_A_ receptor blocker, picrotoxin, confirming that the synaptic current responses were GABAergic. Functional synaptic connections were first detected in MSN-MSN pairs by the induction of post-synaptic currents by evoked pre-synaptic APs. Once connectivity had been established, we examined the synaptic failure rate by inducing APs at low frequency (once every 15 s) and measured whether a post-synaptic current response was induced. The synaptic failure rate did not differ significantly between groups (Figure 7B), suggesting that pre-synaptic release probability is preserved and indicating that baseline functional synaptic MSN–MSN connectivity *in vitro* is maintained. Consistent with this, the amplitude of evoked synaptic currents in connected pairs was not significantly altered, indicating preserved postsynaptic responsiveness (Figure 7C). This is further supported by equivalent responses to exogenous GABA application (Figure S2A), suggesting that postsynaptic GABA_A_ receptor function and quantal response size are not detectably altered in C9-MSNs.

Notably, GABAergic transmission is a key determinant of temporal fidelity in inhibitory microcircuits, governing the precise timing and coordination of neuronal activity.^42^ We therefore investigated whether altered AP dynamics would cause altered temporal delivery of synaptic responses. C9-MSN pairs exhibited significant alterations in the temporal properties of synaptic transmission. Specifically, synaptic latency, the interval between the presynaptic AP peak and the onset of the induced postsynaptic current, was significantly prolonged in C9-MSN pairs compared to controls (Figures 7A and 7D). In addition, trial-to-trial variability of the onset of the synaptic current response (jitter) was more heterogeneous and overall increased (Figures 7A and 7E). These data are consistent with impaired spike initiation precision at the AIS. This suggests that increased synaptic latency and jitter arise, at least in part, from upstream variability in AP generation rather than changes in synaptic release machinery.

Given the contribution of reduced BK channel function to AP repolarization kinetics in C9-MSNs and that the temporal dynamics of APs could be partially rescued using NS11021, we next tested whether enhancement of BK channel function could restore temporal fidelity of synaptic transmission. Application of the BK channel potentiator NS11021 significantly reduced both synaptic latency and jitter in C9-MSN pairs, partially normalizing temporal precision toward control levels (Figures 7A–7D, and 7E). We note that the principal constituent of our intracellular pipette solution contains cesium, a BK channel blocker, thus we do not expect the application of NS11021 to impact our synaptic currents. The increased latency and jitter of synaptic responses in C9-MSN pairs is parsimoniously explained by impaired spike initiation fidelity at the AIS and slowed AP kinetics, which reduce the temporal precision of axonal depolarization reaching presynaptic terminals. Collectively, these data demonstrate that AIS shortening and altered AP waveform dynamics in C9-MSNs degrade the temporal fidelity of inhibitory transmission within MSN microcircuits.

## Discussion

In this study, we used iPSC-derived MSNs to define the evolution and mechanism of intrinsic hypoexcitability in *C9ORF72*^RE^ FTD/ALS striatal neurons. We identify a progressive, MSN-selective reduction in excitability driven primarily by AIS shortening, which limits spike initiation and firing output. In parallel, we observe coordinated reduction of slow-activated Kv conductance’s, including BK and Kv2.1 channels, which contribute to altered spike repolarization dynamics. The directionality of these changes suggests maladaptive, activity-dependent remodeling rather than a primary cause of hypoexcitability. Importantly, these intrinsic abnormalities translate to impaired temporal precision of inhibitory synaptic transmission between MSNs, establishing a functional link between AIS disruption, spike waveform remodeling, and circuit-level dysfunction.

Our findings significantly extend current models of FTD/ALS pathogenesis by implicating human striatal MSNs, neurons recognized for their fundamental role in basal ganglia function, as a previously underappreciated and mechanistically vulnerable population in *C9ORF72*^RE^ FTD/ALS. Importantly, this hypoexcitability phenotype was absent in MNs derived from the same patient iPSC lines (using the Du et al., 2015 protocol) and was fully rescued in isogenic gene-corrected MSNs, supporting both enhanced cell-type vulnerability to the development of hypoexcitability and a direct link to the pathogenic repeat expansion. Our differentiation protocol reliably produced enriched DARPP-32^+^, GABA^+^ MSNs across genotypes, with minimal astrocyte contamination and no evidence of altered cell viability (active caspase-3 staining), further supporting a vulnerability to developing functional hypoexcitability.

Detailed electrophysiological analysis demonstrated that C9-MSNs exhibit a persistent reduction in intrinsic excitability throughout maturation, characterized by an impaired ability to fire multiple APs and a distorted AP waveform. These deficits were not attributable to general developmental abnormalities in maturation, as key subthreshold properties (RMP, capacitance, and input resistance) were unaltered. Similarly, the functional expression of AMPA, GABA_A_, and NMDA receptors, which are tightly regulated during MSN development, remained constant across all patient lines.

Mechanistically, we identified two key post-threshold contributors to the excitability deficits observed in C9-MSNs. First, phase-plane analysis shows a disruption in AP initiation, indicating AIS deficits that were confirmed by ankyrin-G immunostaining, which in turn, revealed a significant reduction in AIS length in C9-MSNs. As the AIS is the principal site of AP initiation due to its high density of NaV channels, structural shortening likely reduces the capacity to initiate APs effectively.^39^ While AIS disruption under physiological stress has been previously reported in ALS MNs,^42^ our findings extend this pathophysiology to striatal MSNs, possibly underscoring AIS dysfunction as a broader hallmark of FTD/ALS-associated neuronal dysfunction. Typically, APs initiate at the AIS and backpropagate to the somatodendritic compartment, the site at which we are making our patch-clamp recordings. In this regard, the phase plane analysis also revealed a substantial reduction in the somatodendritic dV/dt peak and reduced AP amplitude in C9-MSNs. However, somatic voltage-clamp recordings in C9-MSNs showed intact NaV channel expression and function, indicating that the reduced somatodendritic dV/dt peak is not due to NaV dysfunction at the soma. This indicates that impaired functional coupling between the AIS and soma is prominent in C9-MSNs, where reduced AIS AP efficacy leads to insufficient activation of somatic NaV channels to cause a reduced somatodendritic dV/dt peak and overall AP amplitude. Importantly, this in turn likely hinders the recruitment of downstream voltage-gated Kv channels, prolonging depolarization, increasing the risk of NaV channel inactivation, and compounding the overall hypoexcitability. Together, these data position AIS dysfunction as a central driver of impaired AP initiation in C9-MSNs.

Second, C9-MSNs exhibited marked abnormalities in AP repolarization, including prolonged AP duration and a depolarized AHP, consistent with dysfunction in Kv channels. While impaired activation of somatic Kv channels may result partially from AIS-soma decoupling, as previously discussed, voltage-clamp recordings further revealed a significant reduction in slow-activated Kv conductance’s in C9-MSNs, whereas I_A_-Kv currents remained unaffected. Pharmacological analysis identified reduced functional expression of slow-activated BK channels and Kv2.1 channels as contributors to the repolarization deficits. These channels are highly expressed in striatal neurons, particularly in the somatic compartment of MSNs, and are known to shape AP repolarization and AHP dynamics.^35^^,^^36^^,^^37^ Importantly, activation of BK channels using the selective modulator NS11021 partially rescued AP waveform features, confirming a role for BK channel dysfunction in C9-MSNs.

Previously, loss of Kv2.1 and BK channel function has been associated with increased neuronal excitability,^35^ including models of epilepsy and cognitive disorders. However, our findings show that C9-MSNs are hypoexcitable. This apparent discrepancy may reflect the unique cellular context in C9-MSNs: we propose that the reduction in Kv2.1 and BK channels, which are known to be highly plastic in their expression, may represent an inappropriate maladaptive response to reduced excitability in C9-MSNs. However, given that the AIS is the key determinant of AP firing frequency^39^ and that AP initiation is already impaired due to AIS shortening in C9-MSNs, we propose that BK channel potentiation with NS11021, whilst positive for waveform properties in C9-MSNs, fails to enhance firing frequency. We were unable to test the potentiation of Kv2.1 given the lack of selective positive modulators. We propose that in C9-MSNs, the loss of slow-activated Kv function is an inappropriate adaptive response to upstream AIS dysfunction, which ultimately imposes a rate-limiting constraint on neuronal excitability.

Given that striatal computation critically depends on precisely timed inhibitory interactions,^41^ impaired spike timing, rather than loss of connectivity, may represent a key circuit-level consequence of intrinsic neuronal dysfunction in *C9ORF72*-associated disease. Our paired-recording experiments demonstrate that functional connectivity between MSNs is preserved, with unchanged connection probability, synaptic failure rate, and postsynaptic response amplitude. Instead, the dominant defect lies in temporal deficits: prolonged synaptic latency and increased trial-to-trial jitter. These findings indicate that while synaptic release machinery and postsynaptic receptor responsiveness remain intact, the precision with which APs are initiated and propagated to presynaptic terminals is compromised.

Temporal fidelity in inhibitory microcircuits is essential for coordinating ensemble activity and shaping the output of basal ganglia networks.^41^ The observed prolongation of synaptic latency in C9-MSN pairs suggests a systematic delay in axonal depolarization reaching the presynaptic terminal, whereas increased jitter reflects reduced reliability in the timing of spike-triggered neurotransmitter release. Together, these alterations are consistent with impaired spike initiation and propagation dynamics arising from AIS shortening and altered AP waveform properties. Importantly, the absence of changes in synaptic amplitude or failure rate argues against primary defects in vesicle release probability or postsynaptic receptor function, supporting the conclusion that temporal disruption originates upstream of the synapse. The partial rescue of synaptic latency and jitter following BK channel potentiation further reinforces this interpretation. Although NS11021 did not restore overall firing frequency, it sharpened AP waveform characteristics and improved temporal precision of inhibitory transmission.

These findings highlight a potentially underappreciated mode of circuit dysfunction in FTD/ALS, where degradation of temporal coding without overt neuronal loss suggests an early dysfunction in the disease. Such timing defects may precede and potentially contribute to later structural degeneration by destabilizing network activity patterns and impairing coordinated inhibitory control. Our data directly implicate impaired recurrent lateral inhibition between MSNs, which is critical for recurrent inhibition to sculpt population dynamics and regulate downstream basal ganglia output. Furthermore, striatal outputs like the globus pallidus could become disinhibited, thereby altering downstream basal ganglia-thalamocortical circuit activity.^5^^,^^42^ Thus, reduced temporal fidelity could substantially alter information processing even in the absence of major cell loss.

Therapeutically, restoring AP waveform properties is essential for neurotransmitter release. We show that targeting BK potassium channels improves AP waveform dynamics in C9-MSNs, partially restoring synaptic timing. However, given AIS dysfunction and soma-AIS decoupling in C9-MSNs, strategies aimed solely at enhancing excitability via somatic depolarization may be insufficient. Instead, our data indicate that targeting axonal or synaptic mechanisms downstream of the initiation site may offer greater therapeutic potential.

Our findings therefore support a model in which AIS disruption and maladaptive ion channel remodeling in C9-MSNs impair not only intrinsic excitability but also the temporal structure of inhibitory signaling. By linking cellular excitability deficits to measurable alterations in synaptic timing within defined microcircuits, we provide functional evidence that intrinsic membrane abnormalities translate directly into circuit-level dysfunction. This mechanistic bridge between subcellular pathology and network behavior may help explain early striatal involvement in *C9ORF72*-associated FTD/ALS prior to overt neurodegeneration.

### Limitations of the study

This study uses iPSC-derived neurons, which model cell-autonomous disease mechanisms but do not fully capture the aging, multicellular, and circuit-level environment of the adult human striatum. Although striatal pathology in FTD/ALS has been suggested to preferentially involve the ventral striatum,^2^^,^^8^^,^^10^^,^^13^^,^^15^^,^^18^^,^^19^^,^^20^ reliable molecular markers to clearly separate dorsal and ventral MSN populations in human iPSC-derived systems remain limited. As such, our findings are best interpreted as reflecting general MSN dysfunction rather than subregion-specific vulnerability. We identify AIS shortening and altered potassium channel function as contributors to MSN hypoexcitability, although the upstream mechanisms linking the *C9ORF72*^*RE*^ to these changes remain unresolved. Striatal MSNs are now emerging as a key vulnerable cell type within a broader *C9ORF72*-linked gene network associated with selective brain atrophy in FTD/ALS.^43^ In this regard, it will be important to determine whether this extends to sporadic and other genetic FTD/ALS backgrounds. We also highlight that our study has used iPSC lines derived from male participants. Given that striatal excitability is reported as sex-dependent,^44^ we acknowledge that extending the investigation to female participants will be an important step. Despite these limitations, our data support striatal MSN dysfunction as a mechanistically relevant and potentially targetable feature of *C9ORF72*-associated FTD/ALS.

## Resource availability

### Lead contact

Further information and requests for resources and reagents should be directed to and will be fulfilled by the lead contact, Matthew R. Livesey (m.r.livesey@sheffield.ac.uk).

### Materials availability

This study did not generate new unique reagents.

### Data and code availability

• All data reported in this paper will be shared by the lead contact upon request.
• This paper does not report original code.
• Any additional information required to reanalyze the data reported in this paper is available from the lead contact upon request.

## Acknowledgments

The authors acknowledge the support of funding from the Motor Neurone Disease Association (Livesey/Oct20/900-792 to MRL and LF) and philanthropic support to MRL. Graphical abstract created in BioRender. Pasniceanu, I. (2026) https://BioRender.com/61xlga5. Publishing costs were, in part, covered by the University of Sheffield Institutional Open Access Fund.

## Author contributions

Conceptualization, M.R.L. and L.F.; methodology, I.-S.P., M.S.A., C.D.S., T.M., L.F., and M.R.L.; investigation and technical support, I.-S.P., M.S.A., C.D.S., T.M., M.K., C.T., R.J.H.W., L.F., and M.R.L.; writing – original draft, I.-S.P., M.S.A., C.D.S., T.M., D.C.F., R.J.H.W., L.F., and M.R.L.; writing – review and editing, I.-S.P., M.S.A., and M.R.L.; funding acquisition, M.R.L. and L.F.; resources, M.R.L. and L.F.; supervision, C.D.S., L.F., and M.R.L.

## Declaration of interests

The authors declare no competing interests.

## STAR★Methods

### Key resources table

**Table:** 

REAGENT or RESOURCE	SOURCE	IDENTIFIER
**Antibodies**		

βIII-Tubulin anti-Mouse (1:500)	Biolegend	Cat# 801201; RRID AB_2313773
Cleaved Caspase-3 anti-Rabbit (1:200)	Merck Millipore	Cat# AB3623; RRID AB_91556
Nestin anti-Mouse (1:200)	Abcam	Cat# Ab18102; RRID AB_444246
GFAP anti-Rabbit (1:500)	Invitrogen	Cat# 13–0300; RRID AB_2532994
GABA anti-Guinea pig (1:1000)	Abcam	Cat# Ab17413; RRID AB_443865
DARPP-32 anti-Rabbit (1:100)	Abcam	Cat# Ab40801; RRID AB_731843
Pax6 anti-Rabbit (1:500)	Abcam	Cat# Ab5790; RRID AB_305110
MAP2 anti-Rabbit (1:1000)	Synaptic Systems	Cat# 188 003; RRID AB_2281442
Ankyrin-G anti-Mouse (1:200)	Neuromab	Cat# 75–146; RRID AB_10673030
Donkey anti-Rabbit Alexa Fluor 488	Invitrogen	Cat# A21206; RRID AB_2535792
Goat anti-Rabbit Alexa Fluor 568	Invitrogen	Cat# A11036; RRID AB_10563566
Donkey anti-Mouse Alexa Fluor 568	Invitrogen	Cat# A10037; RRID AB_11180865
Goat anti-Guinea pig Alexa Fluor 647	Invitrogen	Cat# A21450; RRID AB_2535867

**Chemicals, peptides, and recombinant proteins**		

Accutase	Sigma-Aldrich	A6964-100ML
B-27 supplement	Gibco	11530536
CHIR99021	Merck Millipore	SML1046-25MG
Dickkopf related protein 1(DKK1)	Peprotech	120–30
DMEM	Gibco	11520416
DMH-1	Merck Millipore	D8946-25MG
Glutamax™	Gibco	35050061
Human Recombinant Brainderived neurotrophic factor(BDNF)	Peprotech	AF-450-02
Knockout DMEM	Gibco	10829018
KnockOut DMEM/F12	Gibco	12660012
Matrigel	Corning	356230
*N*-2 supplement	Gibco	15410294
Neurobasal media	Gibco	11570556
Penicillin/Streptomycin	Lonza	DE17-603 E
Polyornithine	Sigma-Aldrich	P3655-100MG
Purmorphamine (PUR)	Merck Millipore	SML0868-25MG
ReLeSR	StemCell Technologies	05872
Retinoic acid	StemCell Technologies	72264
SB431542	Peprotech	3014193
Y27632 Rock inhibitor	Peprotech	1293823
Compound-E	Tocris	6476
Ciliary neurotrophicfactor (CNTF)	Peprotech	450–13
Insulin-like growthfactor-1 (IGF-1)	Peprotech	100–11
mTeSR™	StemCell Technologies	100–0276
Valproic acid	Merck Millipore	PHR1061-1G
DMSO	Sigma Aldrich	D2650-100mL
Cyanquixaline(CNQX)	Tocris	0190
(2 R)-amino-5-phosphonovaleric acid (APV)	Biogems	7908985
Tetrodotoxin Citrate	Alomone Labs	T-500
Strychnine Hyrdochloride	Sigma Aldrich	S8753
Picrotoxin (PTX)	Sigma Aldrich	P1675
Tetraethylammonium-chloride(TEA)	Merck	T2265
Paxilline	Tocris	2006
Guangxitoxin 1 E	Tocris	5676

**Experimental models: Cell lines**		

Human Control-1 iPSC line	Coriell	GM23338
Human Control-2 iPSC line	Desmarais et al.^26^	MIFF1
Human C9-1 iPSC line	Cedars-Sinai Biomanufacturing Center	CS28iALS-C9nxx
Human C9-2 iPSC line	Cedars-Sinai Biomanufacturing Center	CS29iALS-C9nxx
Human C9-3 iPSC line	Cedars-Sinai Biomanufacturing Center	CS52iALS-C9nxx
Human C9-2Δ iPSC line	Cedars-Sinai Biomanufacturing Center	CS29iALS-C9n1.ISOxx
Human C9-3Δ iPSC line	Cedars-Sinai Biomanufacturing Center	CS52iALS-C9n6.ISOxx

**Software and algorithms**		

OriginPro 2023b	OriginLab	https://www.originlab.com/2023b
WinWCP v5.7.7 and WinEDR V2.7.6	John Dempster, University of Strathclyde	https://spider.science.strath.ac.uk/sipbs/software_ses.htm

**Other**		

Columbus Data Storage and Analysis System	Perkin Elmer	https://www.revvity.com/gb-en/product/image-data-storage-and-analysis-system-columbus
96 well Phenoplate™	Revvity	6055308
Opera Phenix™ High Content Screening System	Revvity	HH25000000
Axon Multiclamp 700 B amplifier	Molecular Devices	700 B
BNC-2090A aquisition interface	National Instruments	BNC-2090A

### Experimental model and study participant details

*Sources of iPSCs*. This study employed human iPSC lines. Full iPSC characterisation, including cell authentication by STR (Short Tandem Repeat) analysis, was performed by the supplier. Lines employed, as previously utilised by our previous studies^29^^,^^31^^,^^45^^,^^46^; Con-1 (GM23338, healthy, Male, age 55, Caucasian, Coriell), Con-2 (MIFF-1; healthy, Male, age neonatal, Caucasian, in house line; see ref. 26), C9-1 (CS28iALS-C9nxx, *C9ORF72*^*RE*^, Male, age 47, Cedars-Sinai Biorepository), C9-2 (CS29iALS-C9nxx, *C9ORF72*^*RE*^, Male, age 47, Cedars-Sinai Biorepository), C9-3 (CS52iALS-C9nxx, *C9ORF72*^*RE*^, Male, age 49, Cedars-Sinai Biorepository), C9-2Δ (CS29iALS-C9n1.ISOxx, isogenic control of C9-2, Cedars-Sinai Biorepository) C9-3Δ (CS52iALS-C9n6.ISOxx, isogenic control of C9-3, Cedars-Sinai Biorepository). iPSC lines used in this study were obtained from commercial or institutional repositories and were derived from donors with informed consent obtained by the originating providers. No new human participants were recruited, and no human subject intervention was performed in this study. All iPSC lines were confirmed to be mycoplasma-free prior to use by the supplier and periodic checks using MycoAlert Mycoplasma Detection Kit (Lonza Bioscience). Experimental groups were defined by genotype. Control groups comprised Con-1, Con-2, C9-2Δ and C9-3Δ lines, while disease groups comprised C9-1, C9-2 and C9-3 *C9ORF72*^*RE*^ lines.

### Method details

*iPSC maintenance*. iPSCs were maintained on Matrigel-coated 6-well plates (0.1 mg/mL in cold KnockOut DMEM, 1 h at room temperature). Cells were cultured in serum-free mTeSR Plus (StemCell Technologies) medium with media changes every 48 h and passaged every 4–6 days at 70% confluency as aggregates using ReLeSR (StemCell Technologies) according to the manufacturer’s instructions.

*Neural precursor cell (NPC) differentiation*. iPSC to NPC differentiation was performed by adapting an already established protocol.^31^ For differentiation to NPCs, iPSCs were transferred to Matrigel-coated plates (0.1 mg/mL, Corning) and grown to 100% confluency. The following day (Day 1), cells were maintained in basal media comprising KnockOut DMEM/F12 and Neurobasal medium (1:1; Gibco) supplemented with 0.5% (v/v) N2 (Gibco), 1% (v/v) B-27 (Gibco), 1% (v/v) GlutaMAX (Gibco), and 1% (v/v) Penicillin/Streptomycin (Lonza), together with 3 μM CHIR99021 (Merck Millipore), 2 μM DMH-1 (Merck Millipore), and 2 μM SB431542 (Peprotech), with daily media changes. From day 7–12, cells were cultured in the same basal media supplemented with 0.1 μM all-trans retinoic acid (StemCell Technologies), 0.5 μM purmorphamine (Merck Millipore), 1 μM CHIR99021, 2 μM DMH-1, and 2 μM SB431542. Between days 7–9, cells were passaged using Accutase (Sigma-Aldrich) and replated onto Matrigel-coated plates in the presence of 10 μM Y27632 ROCK inhibitor (Peprotech), which was removed 24 h post plating. Following day 12, cells were passaged with Accutase and replated onto fresh Matrigel-coated 6-well plates in 10 μM Y27632 ROCK inhibitor for 24 h. The NPCs were maintained in expansion media – basal media supplemented with 3 μM CHIR99021, 2 μM DMH-1, 2 μM SB431542, 0.1 μM all-trans retinoic acid, 0.5 μM purmorphamine, and 0.5 mM valproic acid (Merck Millipore). Media was replaced every 48 h and cells were passaged at 80–100% confluency in the presence of 10 μM Y27632 ROCK inhibitor. NPCs were cryopreserved in 10% DMSO (Sigma-Aldrich) + 90% NPC expansion media, and recovered by rapid thawing followed by replating in NPC expansion media containing ROCK inhibitor for 24 h.

*Differentiation of NPCs into MSNs.* The differentiation of MSNs from NPCs was adapted from a previously established protocol.^23^ When day 12 NPCs reached 100% confluency, cultures were plated, as described above, and were switched to MSN differentiation media containing KnockOut DMEM/F12 and Neurobasal media (1:1) supplemented with 1% (v/v) B-27, 1% (v/v) GlutaMax, 0.5% (v/v) *N*-2, 1% (v/v) Penicillin/Streptomycin, brain-derived neurotrophic factor (BDNF, 30 ng/mL, PeproTech), Dickkopf-1 (DKK-1, 100 ng/mL, PeproTech) and Sonic hedgehog (SHH, 200 ng/mL, PeproTech). On day 20–21, cells were gently dissociated with Accutase. For electrophysiological recordings, sterilised 13 mm HCl-treated glass coverslips were incubated overnight with Poly-L-ornithine (0.1 mg/mL, Sigma-Aldrich), then washed 3 times with PBS before a one-hour incubation with Matrigel (0.1 mg/mL). For electrophysiology recordings, 120,000–150,000 cells were plated per well in a 24-well plate, while 30,000 cells were replated per well on 96-well plates for immunocytochemistry. On the day of plating, the medium was supplemented with Y-27632 (10 μM) and switched to fresh medium the next day. The media was thereafter changed every 48 h. On day 25, cultures were maintained in differentiation media containing Neurobasal, B-27 supplement (2% v/v), penicillin/streptomycin (1% v/v) and BDNF (50 ng/mL) until use.

*Differentiation of NPCs into MNs.* The differentiation of MNs from NPCs was adapted from a previously established protocol.^31^ NPCs were cultured to ∼100% confluency in NPC expansion media before induction of MN differentiation. From day 13–18, cells were cultured in MN induction medium comprising basal media containing KnockOut DMEM/F12 and Neurobasal media (1:1) supplemented with 0.5% (v/v) N2, 0.5% (v/v) B-27, 1% (v/v) GlutaMAX, 1% (v/v) Penicillin/Streptomycin, 0.5 μM all-trans retinoic acid, and 0.1 μM purmorphamine, with media changes every 48 h. From day 19–28, cells were maintained in MN differentiation medium consisting of the same basal media supplemented with 0.5 μM RA, 0.1 μM purmorphamine, 0.1 μM Compound-E (Tocris), 10 ng/mL BDNF, and 10 ng/mL insulin-like growth factor-1 (IGF-1; PeproTech), with media changes every 48 h. On days 20–21, cells were gently dissociated using Accutase, counted, and replated onto plates pre-coated with Poly-L-ornithine (0.1 mg/mL for 1 h at room temperature with 3 subsequent washes in dH_2_O), followed by Matrigel as previously described, in the presence of 10 μM Y-27632 ROCK inhibitor. For immunocytochemistry and electrophysiology experiments, cells were plated as described for MSNs. Fresh medium was added 24 h after replating, and media was subsequently changed every 48 h. From day 29–40, cells were transitioned to MN maturation medium consisting of Neurobasal medium supplemented with 2% (v/v) B-27, 1% (v/v) Penicillin/Streptomycin, 10 ng/mL BDNF, 10 ng/mL ciliary neurotrophic factor (CNTF; PeproTech), and 10 ng/mL IGF-1, with media changes every 48 h until use.

*Immunocytochemistry*. Cultures on optically transparent 96-well Phenoplate (Revvity) were fixed in 3.8% PFA at room temperature for 10 min. Cells were permeabilised with 0.3% Triton X-100, blocked with 5% donkey serum and incubated overnight at 4°C with primary antibodies against β-III-tubulin (dilution 1:500, Biolegend), active caspase-3 (dilution 1:200, Millipore), Nestin (dilution 1:200, Abcam), GFAP (1:500, Invitrogen), GABA (dilution 1:1000, Abcam), DARPP-32 (dilution 1:100; Abcam), PAX6 (dilution 1:500, Abcam), MAP2 (dilution 1:1000, Synaptic Systems) and Ankyrin-G (dilution 1:200, NeuroMab). Cells were washed 3× using PBS, then incubated with Alexa Fluor secondary antibodies (dilution, 1:400; Invitrogen) for 1 h at room temperature. Cells were washed an additional 3× using PBS and imaged using an Opera Phenix High Content Screening System (Revvity). Z-stacks of 7 or more planes were separated by 0.5 μm and obtained from a minimum of 7 fields per well from at least three technical replicates per experiment (unless otherwise stated). All image files were processed via Columbus Data Storage and Analysis System (PerkinElmer). AIS length and distance away from the center of the cell soma were analyzed using FIJI.

*Patch-clamp electrophysiology*. For single-patch electrophysiological experiments, whole-cell patch-clamp recordings^47^^,^^48^^,^^49^ were performed using an Axon Multiclamp 700 B amplifier (Molecular Devices, Union City, CA). Coverslips containing cultured neurons were transferred to a recording chamber continuously perfused with extracellular solution containing (in mM): 152 NaCl, 2.8 KCl, 10 HEPES, 2 CaCl_2_, and 10 glucose (pH 7.3, 320–330 mOsm) at room temperature (20°C–23°C) using a gravity-fed system. Patch pipettes (4–7 MΩ) were filled with a K-gluconate-based internal solution containing (in mM): 155 K-gluconate, 2 MgCl_2_, 10 Na-HEPES, 10 Na-phosphocreatine, 2 Mg-ATP, and 0.3 Na_3_-GTP (pH 7.3, 300 mOsm). For postsynaptic recordings in paired experiments, pipettes (3–6 MΩ) were filled with a CsCl-based internal solution containing (in mM): 130 CsCl, 10 NaCl, 2 MgCl_2_, 0.5 EGTA, 10 HEPES, 2 Na_2_-ATP, 0.03 Na-GTP, and 0.2 QX-314 (pH 7.2, adjusted with CsOH). The high intracellular chloride allowed GABAergic currents to be recorded as inward currents at −70 mV. For current-clamp recordings, neurons were recorded in the presence of synaptic blockers (CNQX 5 μM, D-APV 50 μM, picrotoxin 50 μM, and strychnine 20 μM) using bridge balance mode with pipette capacitance neutralisation. Paired recordings were performed between neurons with somatic separation ≤50 μm, with the presynaptic neuron recorded in current-clamp and the postsynaptic neuron in voltage-clamp at −70 mV. To facilitate identification of synaptically coupled pairs, the postsynaptic neuron was first screened for functional synaptic input using hypertonic sucrose (0.2 M sucrose added to the extracellular solution), which evokes neurotransmitter release from nearby presynaptic terminals.^50^ Only postsynaptic neurons exhibiting clear sucrose-evoked synaptic responses were selected for subsequent paired-recording experiments. Series resistance was compensated by 80% in voltage-clamp and continuously monitored; experiments were terminated if series resistance exceeded 25 MΩ or changed by >20%. Signals were low-pass filtered at 2 kHz and digitised at 10 kHz via a BNC-2090A interface (National Instruments) and acquired using WinWCP v5.7.7 and WinEDR V2.7.6 software (J. Dempster, University of Strathclyde). All membrane potential values, except amplitude measurements, were corrected for a −14 mV liquid junction potential.

*Electrophysiological parameter quantification*: Input resistance was calculated from the steady-state voltage deflection in response to small hyperpolarising current injections (typically −20 pA) using Ohm’s law (R_IN_ = ΔV/ΔI), measured in the linear subthreshold range. Resting membrane potential was defined as the baseline membrane voltage recorded immediately after achieving whole-cell configuration in current-clamp mode, in the absence of injected current (I = 0 pA). Membrane capacitance was estimated from the capacitive transients elicited by small voltage steps under voltage-clamp conditions, calculated from the integral of the capacitive current. Action potential threshold was defined as the membrane potential at which the first derivative of voltage (dV/dt) exceeded 5 mV/ms. Action potential amplitude was measured as the voltage difference between threshold and the peak of the action potential. Half-width was defined as the duration of the action potential measured at 50% of peak amplitude, calculated between the rising and falling phases of the spike.

Afterhyperpolarisation was defined as the most negative membrane potential reached following an action potential, measured relative to resting membrane potential, and quantified as peak AHP amplitude. Phase plots were generated by plotting the first derivative of membrane potential (dV/dt) against membrane potential (V) for individual action potentials.

*Leak subtraction protocols (ion channel isolation)*. For NaV currents, cells were held at −70 mV and depolarised to +30 mV to evoke NaV-mediated inward currents. The protocol was then repeated in the presence of tetrodotoxin [TTX (300 nM, Alomone Labs)] to block NaV channels. TTX-sensitive currents were obtained by subtracting the TTX-insensitive current trace from the control trace. Current amplitudes were determined from the peak inward current. Transient A-type K^+^ (I_A_) currents were isolated using a two-step protocol in the presence of TTX. First, I_A_ channels were first primed by stepping the membrane potential from −70 mV to −100 mV for 500 ms, followed by a depolarising step to +35 mV for 400 ms to activate I_A_. A second protocol in which I_A_ was inactivated using a pre-step to −20 mV (500 ms) prior to depolarisation to +35 mV was used to generate a non-I_A_ control trace approximately 15 s after the pre-pulse. I_A_ -specific currents were obtained by subtracting the inactivated trace from the activated trace. slow-activated Kv currents were isolated using pharmacological subtraction in the presence of TTX. Currents were evoked by depolarisation from −70 mV to +30 mV, and recordings were repeated in the presence of tetraethylammonium [TEA (30 mM, Merck) where extracellular NaCl was osmotically reduced to 120 mM]. TEA-sensitive currents were obtained by subtracting TEA-insensitive currents from control recordings. Current amplitudes were measured in the steady-state phase at 225 ms. Specific slow-activated Kv currents mediated by BK and Kv2.1 channels were isolated as described for TEA-sensitive currents though TEA was exchanged for paxilline (10 μM, Tocris) and Guangxitoxin 1 E (100 nM, Tocris), respectively. Current density for each ion channel was determined by dividing the current amplitude by the whole-cell capacitance value for each individual cell.

### Quantification and statistical analysis

*Statistical analysis*. Statistical analyses were performed using Microcal Origin (OriginLab). Data are presented as mean ± standard error of the mean (SEM). In all experiments, *n* refers to the number of individual cells, and *N* denotes the number of independent iPSC differentiations (*de novo* preparations). Datasets were generally pooled from at least three *de novo* differentiations unless otherwise stated. Statistical comparisons were performed using unpaired or paired Student’s t-tests, one-way ANOVA with Tukey’s post hoc correction, or two-way ANOVA, as appropriate for dataset structure. *p*-values <0.05 were considered statistically significant and are denoted as ∗*p* < 0.05, ∗∗*p* < 0.01 and ∗∗∗*p* < 0.001. Statistical details for individual experiments, including the statistical test used, exact values of n and N, and significance levels, are provided in the corresponding figure legends. Where appropriate, we include source data, as defined within the figure legend used to derive the presented mean ± SEM.

## Supplemental information

Document S1. Figures S1–S5

Document S2. Article plus supplemental information
