# Supplementary material for: Striatal neuron dysfunction in C9ORF72-FTD/ALS is driven by AIS and potassium channel dysregulation
Source: Cell Rep. 2026 Jul 7;45(7):117672. doi: 10.1016/j.celrep.2026.117672 (PMC13415681; doi:10.1016/j.celrep.2026.117672)
Supplement: Document S1. Figures S1–S5 [file mmc1.pdf]

Cell Reports, Volume *45*

Supplemental information

Striatal neuron dysfunction

in C9ORF72-FTD/ALS is driven by

AIS and potassium channel dysregulation

Iris-Stefania Pasniceanu, Manpreet S. Atwal, Cleide Dos Santos Souza, Tobias Moll, Marianne King, Connie Treanor, Daniel Cabezas de la Fuente, Ryan J.H. West, Laura Ferraiuolo, and Matthew R. Livesey

SUPPLEMENTARY FIGURES

FIGURE S1

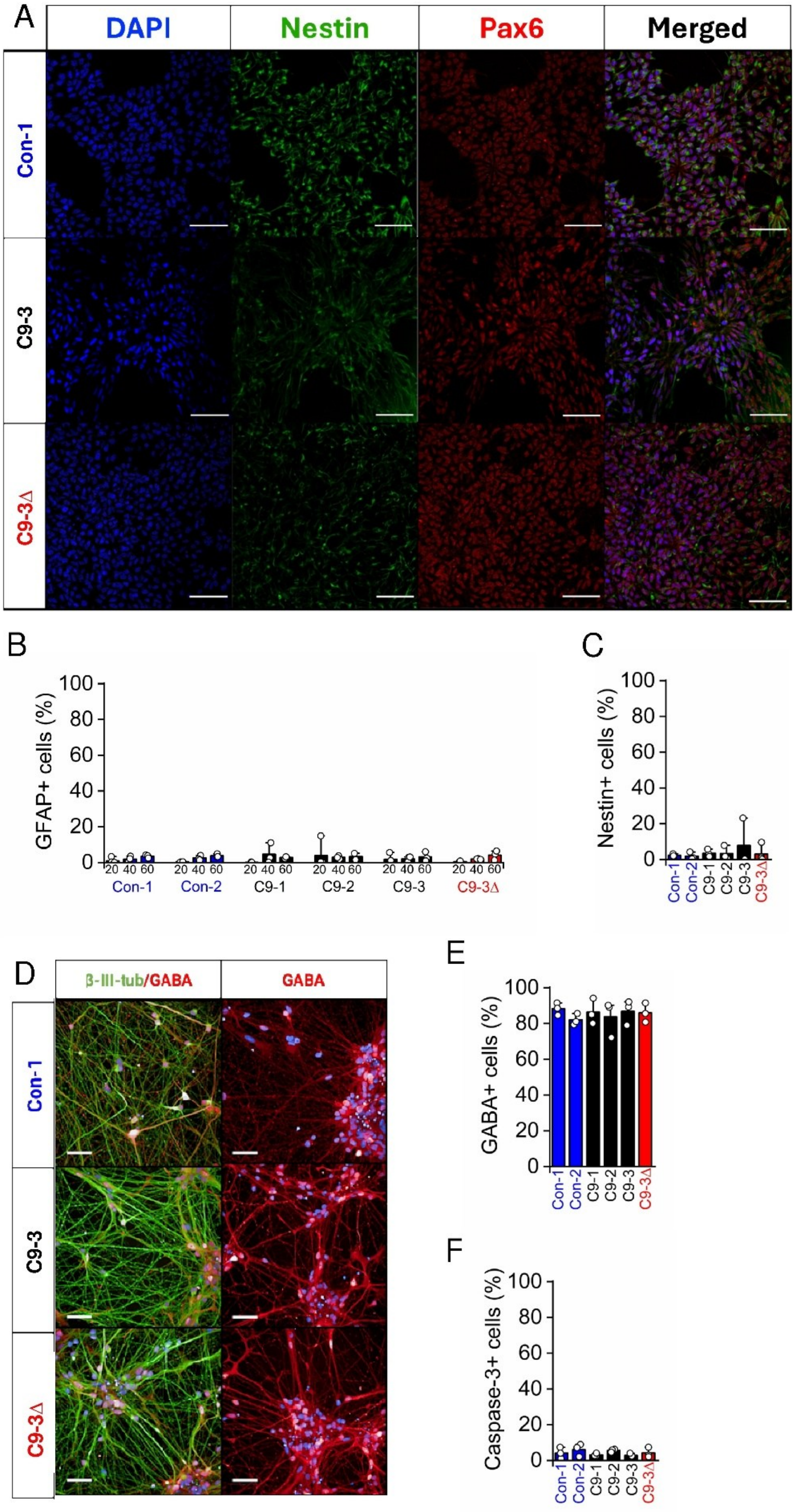

Figure S1*. In vitro* MSN specification. (A). Representative images of NPCs derived from Con-1, C9-3 and C9-3Δ iPSCs stained for NPC markers nestin and Pax6. Nuclei were counterstained with DAPI. Scale bar: 50 µm. (B). Mean±SEM percentage of DAPI labelled cells that were GFAP+ at DIV20-60. Data were derived from at least 3 de novo preparations. (C). Mean±SEM percentage of DAPI cells that were positive for nestin at DIV40. (D). Representative images of DIV60 neurons derived from Con-1, C9-3 and C9-3Δ iPSCs stained for markers β-III-tubulin, GABA and DAPI (left) or GABA and DAPI alone. Scale bar: 50 µm. (E), Mean±SEM percentage of β-III-tubulin+ cells that were positive for GABA at DIV60. Data were derived from at least 3 de novo preparations. (F). Mean±SEM percentage of DAPI cells that were positive for active caspase-3 at DIV60. All data were derived from at least 3 de novo preparations. Statistics, one-way ANOVA followed by Tukey's multiple comparison test.

FIGURE S2

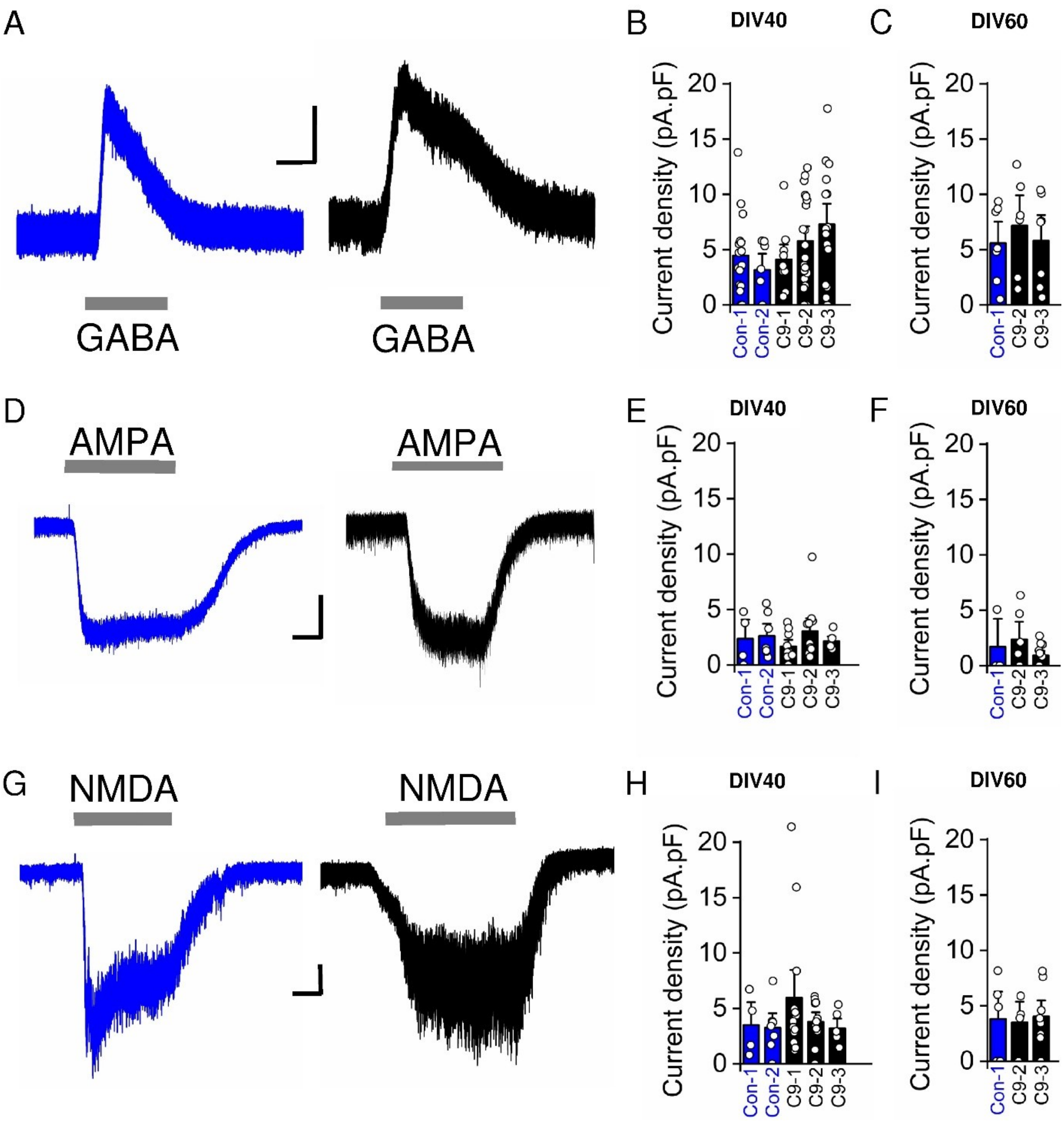

Figure S2. Responses of MSNs to agonists of transmitter-gated ion channels. (A). Representative whole-cell recordings of currents evoked by bath-applied GABA (100μM) from a holding potential of –54mV. Currents were blocked by $GABA_A$ receptor antagonist bicuculline (30μM, data not shown). Scale bar (for all traces), 20 pA, 10ms. (B and C). Mean±SEM whole-cell $GABA_AR$ current density (current amplitude/whole-cell capacitance) for DIV40 and 60, respectively. No significant change in functional current density is observed. Data: Con-1: DIV40, n=17, N= 4; DIV60, n=7, N=2; Con-2: n=7, N=2; C9-1: n=10, N=2; C9-2: DIV40, n=20, N=5; DIV60, n=6, N=2; C9-3: DIV40, n=16, N=3; DIV60, n=7, N=2. (D). Representative whole-cell recordings of currents evoked by bath-applied AMPA (50μM) from a holding potential of –74mV. Currents were blocked by AMPA receptor antagonist CNQX (15μM, data not shown). (E and F). Mean±SEM whole-cell AMPA receptor current density for DIV40 and 60, respectively. No significant change in functional current density is observed. Data: Con-1: DIV40, n=*4*, N=2; DIV60, n=*3*, N=*1*; Con-2: n=7, N=2; C9-1: n=10, N=*3*; C9-2: DIV40, n=*11*, N=*3*; DIV60, n=6, N=*1*; C9-3: DIV40, n=6, N=*2*; DIV60, n=*12*, N=2. (G). Representative whole-

cell recordings of currents evoked by bath-applied NMDA (100μM, in the presence of glycine, 50μM) from a holding potential of –74mV. Currents were blocked by NMDA receptor antagonist APV (50μM, data not shown). (H and I). Mean±SEM whole-cell NMDA receptor current density for DIV40 and 60, respectively. No significant change in functional current density is observed. Data: Con-1: DIV40, n=4, N=2; DIV60, n=5, N=1; Con-2: n=7, N=3; C9-1: n=13, N=3; C9-2: DIV40, n=11, N=3; DIV60, n=4, N=2; C9-3: DIV40, n=6, N=2; DIV60, n=8, N=2. Statistics, one-way ANOVA followed by Tukey's multiple comparisons test.

FIGURE S3

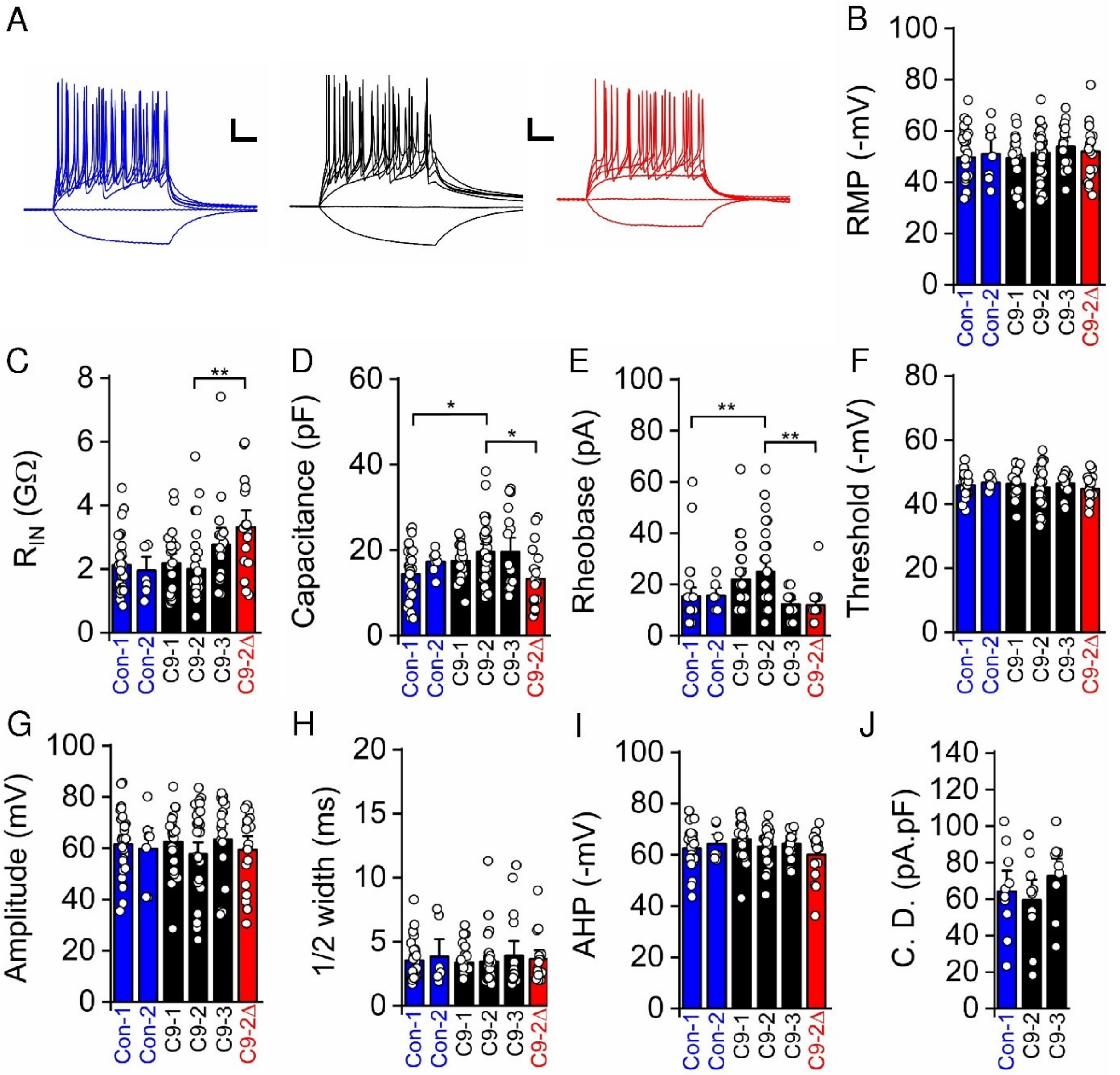

Figure S3. iPSC-derived motor neuron excitability. (A). Representative whole-cell current-clamp voltage responses from individual Con-1, C9-2 and C9-2Δ MNs at DIV40 are shown in response to a train of incremental current injections (-20pA to 50pA in 10pA steps) from a holding potential held of -84mV. MNs show equivalent excitability properties across all lines. Note that not all voltage responses from the current protocol are shown for clarity. Scale bar, 10 mV, 50 ms. (B-D). Intrinsic membrane property data from DIV40 Con-1, Con-2, Con-3, C9-1, C9-3, C9-2 and C9-2Δ MNs. Mean±SEM resting membrane potential (RMP), input resistance (R_{IN}) and whole-cell capacitance, respectively. Intrinsic membrane properties are not consistently altered between healthy control, C9 and isogenic controls. Data: Con-1: n=29, N=7; Con-2: n=7, N=2; C9-1: n=24, N=7; C9-2: n=33, N=9; C9-3: n=18, N=5; C9-2Δ: day 40, n=18, N=4. (E-I). Key parameters of AP structure are unaltered in DIV40 MNs derived from the same C9 iPSCs. Measurements of the recruitment current required to evoke the first AP (rheobase), threshold potential, AP amplitude, AP duration (half-width) and AHP. All data presented as Mean (±SEM). Statistics, * $p<0.05$, ** $p<0.01$, from one-way ANOVA followed by Tukey's multiple comparisons test. Data: Con-1: n=29, N=7; Con-2: n=7, N=2; C9-1: n=23, N=7; C9-2: n=32, N=9; C9-3: n=17, N=5; C9-2Δ: n=18, N=4. Statistics, * $p<0.05$, ** $p<0.01$,

**** $p<0.0001$, one-way ANOVA followed by Tukey's multiple comparisons test. (J) Mean±SEM current density (C.D.) shows that functional expression of BK channels is not altered in C9-3 *versus* Con-2 and Con-3 MNs. Data: Con-1, n=10, N=2; C9-2, n=10, N=2; C9-3, n=10, N=2. Statistics, one-way ANOVA followed by Tukey's multiple comparisons test

FIGURE S4

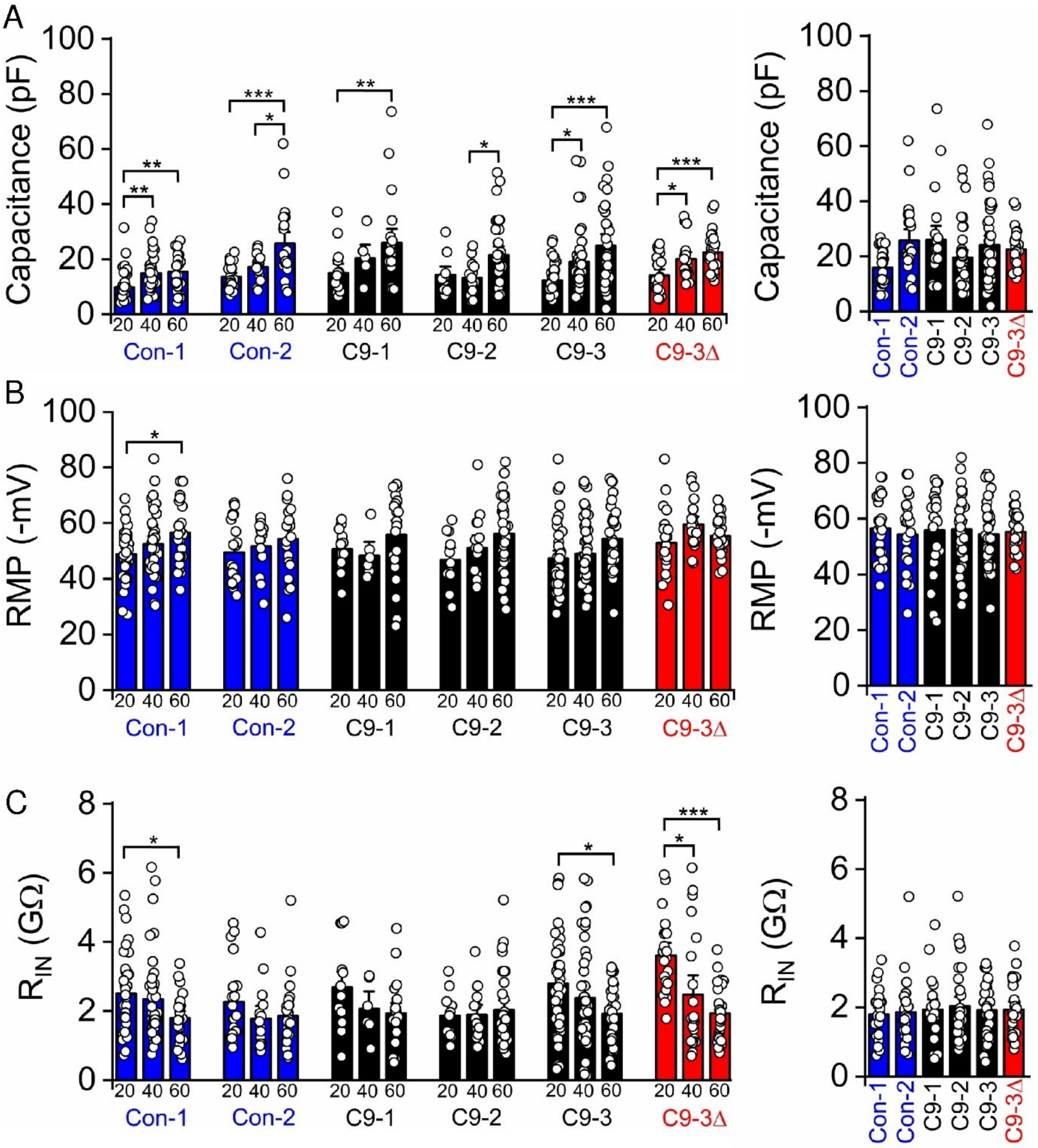

Figure S4. iPSC-derived MSN sub-threshold membrane properties. (A). Mean±SEM whole-cell capacitance data for each MSN line over time from DIV20, 40 and 60 (*left*). Comparative data for DIV60 across each line (*right*). A significant trend towards increased capacitance with culture time is observed across all MSN lines, but comparatively, capacitance measurements across all lines at DIV60 are not different. (B). Data presented as in A, but for resting membrane potential (RMP). The RMP becomes increasingly hyperpolarised with culture time for each line and is not significantly different across all lines at DIV60. (C). Data presented as in A, but for input resistance (R_{IN}). R_{IN} decreases with culture time for each line and is insignificantly different across all lines at DIV60. Data: Con-1: DIV20, n=38, N=14; DIV40, n=34, N=9; DIV60, n=30, N=11; Con-2: DIV20, n=21, N=4; DIV40, n=15, N=10; DIV60, n=21, N=6; C9-1: DIV20, n=15, N=3; DIV40, n=6, N=2; DIV60, n=32, N=6; C9-2: DIV20, n=19, N=5; DIV40, n=16, N=4; DIV60, n=30, N=7; C9-3: DIV20, n=41, N=11; DIV40, n=39, N=11;

DIV60, n=41, N=12; C9-3Δ: DIV21, n=20, N=9; DIV40, n=20, N=8; DIV60, n=26, N=8. Statistics, * $p<0.05$, ** $p<0.01$, *** $p<0.001$, **** $p<0.0001$ from one-way ANOVA followed by Tukey's multiple comparisons test.

FIGURE S5

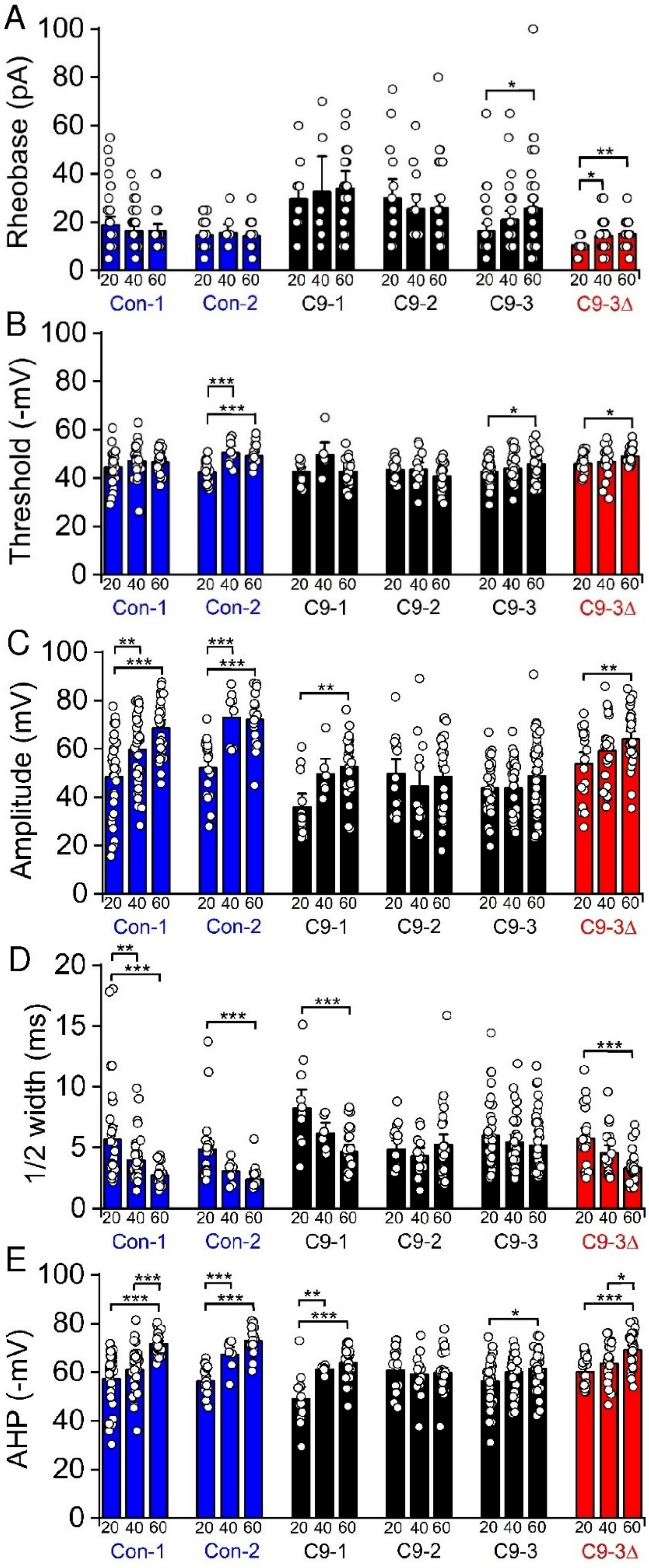

A
Rheobase (pA)
B
Threshold (-mV)
C
Amplitude (mV)
D
1/2 width (ms)
E
AHP (-mV)
20 40 60
Con-1
Con-2
C9-1
C9-2
C9-3
C9-3Δ

Figure S5. MSN AP waveform properties over time. (A-E). Mean±SEM rheobase, threshold, amplitude, half-width and AHP measurements, respectively, at DIV20-60 for all MSN lines investigated. Data shows that the AP waveform impairments of C9-MSNs persist over time. Data: Con-1: n=29-32, N=9-14; Con-2: n=15-21, N=4-10; C9-1: n=5-23, N=2-6; C9-2: n=13-24, N=4-7; C9-3: n=33-36, N=11-12; C9-3Δ: n=20-26, N=8-9. Statistics, * p<0.05, ** p<0.01, *** p<0.001, **** p<0.001 from one-way ANOVA followed by Tukey's multiple comparisons test.
